# HMGCS1 drives cholesterol-dependent membrane repair and shields tumor cells from lymphocyte attack

**DOI:** 10.1038/s41467-026-74022-y

**Published:** 2026-06-05

**Authors:** Yajuan Zhang, Siyao Wang, Tianhang Luo, Haitang Yang, Yongqiang Wang, Tong Rong, Miaowen Zou, Qizhen Fei, Zhonggang Shi, YiFei Zhu, Xin Zhou, Hong Gao, Yun Zhao, Zhengjiang Zhu, Huiyong Yin, Guangchuan Wang, Chenqi Xu, Xiujuan Qu, Feng Yao, Weiwei Yang

**Affiliations:** 1 Key Laboratory of Multi-cell Systems, Shanghai Key Laboratory of Molecular Andrology, CAS Center for Excellence in Molecular Cell Science, Shanghai Institute of Biochemistry and Cell Biology, University of Chinese Academy of Sciences, Chinese Academy of Sciences, Shanghai, China; 2 Shanghai Institute of Thoracic Oncology, Shanghai Chest Hospital, Shanghai Jiao Tong University School of Medicine, Shanghai, China; 3 Department of Gastrointestinal Surgery, Changhai Hospital, Naval Medical University, Shanghai, China; 4 Department of Thoracic Surgery, Shanghai Chest Hospital, Shanghai Jiao Tong University School of Medicine, Shanghai, China; 5 Frontier Medical Center, Tianfu Jincheng Laboratory, Chengdu, China; 6 Department of Pancreatic Surgery, Fudan University Shanghai Cancer Center, Shanghai, China; 7 Department of Oncology, Shanghai Medical College, Fudan University, Shanghai, China; 8 Interdisciplinary Research Center on Biology and Chemistry, Shanghai Institute of Organic Chemistry, Chinese Academy of Sciences, Shanghai, China; 9 Department of Biomedical Sciences, College of Biomedicine, Institute of Digital Medicine, Tung Biomedical Science Center, The Shenzhen Research Institute and Futian Research Institute, City University of Hong Kong, Hong Kong, China; 10 Department of Medical Oncology, the First Hospital of China Medical University, Shenyang, China; 11 Key Laboratory of Systems Health Science of Zhejiang Province, School of Life Science, Hangzhou Institute for Advanced Study, University of Chinese Academy of Sciences, Hangzhou, China; 12 Shanghai Academy of Natural Sciences (SANS), Shanghai, China

**Keywords:** Cancer metabolism, Tumour immunology

## Abstract

Cytotoxic lymphocytes use perforin to form plasma membrane (PM) pores in tumor cells, thereby enabling granzyme-mediated cell death. However, whether and how tumor metabolism enables PM repair to evade immunity is unclear. In this study, using a functional screen targeting 111 metabolic enzymes, we identified hydroxymethylglutaryl-CoA synthase 1 (HMGCS1) as critical for repairing perforin-induced PM damage. HMGCS1 promotes PM repair by initiating de novo cholesterol synthesis, enhancing tumor cell resistance to lymphocyte-mediated killing and impairing the efficacy of NK, CAR-T, and anti-PD-1-based immunotherapies. Beyond its structural role, cholesterol directly binds charged multivesicular body protein 4b (CHMP4B) to enhance its PM localization, facilitating PM repair. Furthermore, oncogenic activation, cytokine, and hypoxia induce c-Jun activation, up-regulating HMGCS1 expression. In lung cancer patients, elevated c-Jun activation, HMGCS1 expression, cholesterol content and PM CHMP4B correlate with reduced anti-PD-1 immunotherapy efficacy. Our findings reveal a tumor immune evasion mechanism wherein HMGCS1 drives cholesterol-dependent PM repair by activating the cholesterol synthesis. Targeting HMGCS1 enhances the effectiveness of immunotherapies.

## Abstract (web summary)

A key mechanism of immune mediated cytotoxic attack against cancer cells relies on perforin induced plasma membrane damage. This study identifies HMGCS1 driven cholesterol synthesis as a membrane repair pathway that enables tumor cell resistance to lymphocyte killing.

## Introduction

Immunotherapy has emerged as a promising and effective approach to treating tumors, complementing traditional approaches such as surgery, chemotherapy, and radiotherapy, thereby revolutionizing cancer treatment^1^. However, the presence of multiple and complex immune evasion mechanisms, such as the absence of tumor-specific antigens, the recruitment of immunosuppressive cells, and the enhancement of resistance to immune cytotoxicity, limits the number of patients who can benefit from immunotherapy^2–5^. Deciphering immune evasion mechanisms is crucial for discovering novel immunotherapy strategies and enhancing the efficacy of existing cancer immunotherapies, thus extending their benefits to a broader patient population.

Destruction of tumor cells depends on the action of cytotoxic T lymphocytes (CTLs) and natural killer (NK) cells. These cells primarily use perforin-granzyme-dependent granule exocytosis to kill tumor cells^6,7^. During this process, perforin and granzymes are released into the immune synapse, leading to the formation of transmembrane pores and the entry of granzyme into target cells^8^. Granzymes induce apoptosis in tumor cells by activating caspases or other apoptotic pathways^9^. Accordingly, tumor cells have evolved mechanisms to resist immune cell killing, such as the activation of multiple anti-apoptotic pathways^10,11^ or the initiation of autophagy to degrade granule enzymes^12,13^. Recently, research has highlighted the significance of the endosomal sorting complex required for transport (ESCRT)-dependent membrane repair system in enhancing tumor cell resistance to immune-mediated killing. This system functions by preventing the formation of perforin-induced pores in the plasma membrane (PM), thereby protecting tumor cells from lysis^7,14^, underscoring the importance of further exploring membrane repair mechanisms as potential targets for improving the efficacy of immunotherapies.

Metabolic reprogramming, a key hallmark of cancer, has been shown to promote tumor immune evasion through multiple mechanisms. First, the enhanced fermentation leads to decreased expression of the MAPK ERK5 to downregulate the expression of major histocompatibility complex (MHC) to evade recognition by immune cells^2^. Besides, boosted NAD^+^ metabolism, as well as increased acetate uptake, activates IRF1 or c-Myc to upregulate the expression of the surface molecule PD-L1 to inhibit T cell function^15,16^. In addition, the expression of anti-apoptotic/pro-survival genes is upregulated by reprogrammed glucose metabolism to resist immune cytotoxicity^4,17^. Moreover, tumor cell-secreted lactate rewires CD8^+^ T cell metabolism to inhibit CD8^+^ T cell cytotoxicity; meanwhile, tumor cell-derived lactate generates intermediates that support the proliferation of immunosuppressive cells^18,19^. However, whether or how metabolism is rewired to support PM repair in tumor cells during lymphocyte killing, thereby promoting tumor immune evasion, remains to be elucidated.

In this study, we performed a high-throughput functional screen using siRNAs targeting 111 key metabolic enzymes and identified hydroxymethylglutaryl-CoA synthase 1 (HMGCS1) as essential for Streptolysin O (SLO)-induced PM damage repair in tumor cells. HMGCS1-dependent de novo cholesterol synthesis enhances PM repair following damage by T or NK cells by producing cholesterol that not only functions as the signal molecule but also as the structural components of the membrane, thereby conferring resistance of tumor cells to lymphocyte killing.

## Results

### Functional screen identifies HMGCS1 necessary for PM repair

**Fig. 1:**
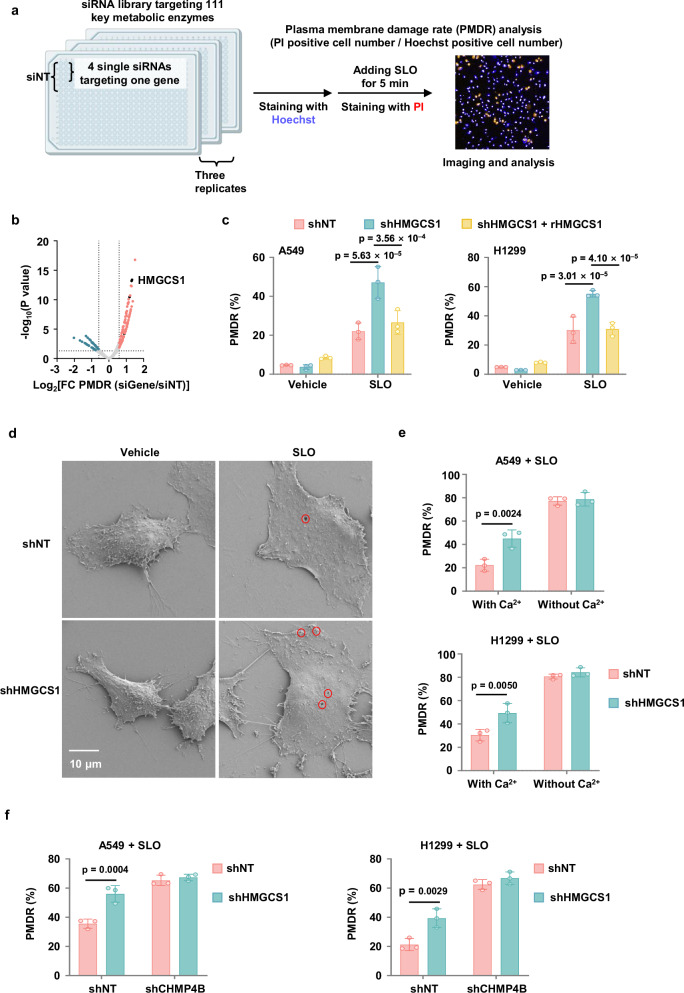
Functional screen identifies HMGCS1 necessary for PM repair. **a**, **b** A549 cells were transfected with individual siRNAs targeting 111 key metabolic enzymes (four siRNAs per gene), or a non-targeting siRNA (siNT) as the negative control. The cells were stained with Hoechst for 20 min, before treated with 0.1 ng/μl SLO and PI for 5 min. Plasma membrane damage rate (PMDR) was measured using the Ensight high-throughput screening system. A schematic diagram of the screening strategy is shown in panel (**a**). Data are presented as a volcano plot from three biological replicates (b, two-tailed Student’s t-test). siRNAs with P < 0.05 (-log_10_P > 1.3) and a PMDR ratio (normalized to the non-targeting siRNA) of <0.67 (-log_2_Foldchange > 0.585, cyan dot) or > 1.5 (log_2_Foldchange > 0.585, red dot) were considered effective. Black: HMGCS1 siRNA 1-4. The 384-well plate in the schematic diagram was created with BioGDP.com^43^. **c** HMGCS1-depleted A549 or H1299 cells were rescued with rHMGCS1 WT. The cells were treated with or without SLO for 5 minutes at 37 °C. PI and Hoechst staining were performed, and PMDR was calculated. **d** HMGCS1 was depleted in A549 cells, which were then treated with or without SLO for 5 minutes at 37 °C. After treatment, the cells were washed, fixed in 2% glutaraldehyde, and subjected to Scanning Electron Microscopy (SEM) imaging analyses. Red circles indicate SLO-induced pores. **e** HMGCS1 was depleted in A549 or H1299 cells, which were treated with or without SLO in PBS containing Ca²⁺ or in PBS free of Ca²⁺. **f** HMGCS1-depleted A549 or H1299 cells were further depleted of CHMP4B. The cells were treated with or without SLO for 5 minutes at 37 °C. PI and Hoechst staining were performed, and PMDR was calculated. **b** Data represent the mean of three biological replicates. **c**, **e**, **f** Data represent the mean ± s.d. of three biologically independent experiments. **d** Data are representative of three independent experiments. **b** Two-tailed Student’s t-test. **c** Two-way ANOVA with Tukey’s multiple comparisons test. **e**, **f** Two-way ANOVA with Sidak’s multiple comparisons test. Also see Supplementary Fig. 1. Source data are provided as a Source Data file.

To identify the metabolic enzyme that supports PM repair of tumor cells, we used short interfering RNAs (siRNAs) to deplete 111 key metabolic enzymes (Supplementary Table 1) in human non-small-cell lung cancer (NSCLC) A549 cells, followed by the treatment of Streptolysin O (SLO) (cytolytic toxin that shares similar protein structure, function, and mode of action with perforin) for 5 min. We stained the cells with Hoechst and propidium iodide (PI) to calculate PM damage rate (PMDR, PI/Hoechst ratio) of these cells. As shown in Fig. 1a, b, the depletion of hydroxymethylglutaryl-CoA synthase 1 (HMGCS1), the enzyme catalyzing the first step of de novo cholesterol synthesis, greatly elevated PM damage with all four independent siRNAs. We then depleted HMGCS1 in A549 or H1299 cells using short hairpin RNA (shRNA) and rescued these cells with shRNA-resistant (r) HMGCS1, and confirmed that HMGCS1 depletion aggravated SLO-induced PM damage, while rescued expression of rHMGCS1 fully recovered the membrane integrity to control cells, excluding the off-targeting possibility of HMGCS1 shRNA (Fig. 1c and Supplementary Fig. 1a). Consistently, overexpression of HMGCS1 reduced the SLO-induced membrane damage in A549 and H1299 cells (Supplementary Fig. 1b, c). Importantly, HMGCS1 depletion did not affect cell death within 5 minutes after SLO treatment (Supplementary Fig. 1d). Moreover, Scanning Electron Microscopy (SEM) imaging analyses confirmed that HMGCS1 depletion aggravated SLO-induced PM damage (Fig. 1d). Notably, HMGCS1 depletion did not affect the viability and proliferation of A549 and H1299 cells (Supplementary Fig. 1e, f). These results indicate that HMGCS1 is required to maintain membrane integrity during SLO treatment.

The influx of Ca^2+^ through the damaged PM into cells has been identified as the key trigger to activate an effective membrane repair response^20^. To ascertain whether HMGCS1 participates in PM damage repair to sustain PM integrity, we treated tumor cells with SLO in PBS buffer without the addition of Ca^2+^ to prevent membrane repair. As shown in Fig. 1e, HMGCS1 depletion markedly increased PM damage in the cells cultured with Ca^2+^, but failed to do so in the cells cultured without Ca^2+^, suggesting that HMGCS1 is required for membrane integrity by regulating membrane repair. The endosomal sorting complex required for transport (ESCRT) complex is one of the main repair complexes involved in membrane repair. To further confirm that HMGCS1 sustains membrane integrity via membrane repair mechanism, we depleted charged multivesicular body protein 4b (CHMP4B), the core component of the ESCRT complex, in A549 or H1299 cells (Supplementary Fig. 1g). PI and Hoechst staining analyses showed that CHMP4B depletion completely abrogated HMGCS1 depletion-enhanced PM damage in the cells after SLO treatment (Fig. 1f). These results demonstrate that HMGCS1 promotes PM repair to sustain membrane integrity in the cells upon SLO treatment.

### HMGCS1-dependent de novo cholesterol synthesis supports PM repair by producing cholesterol

**Fig. 2:**
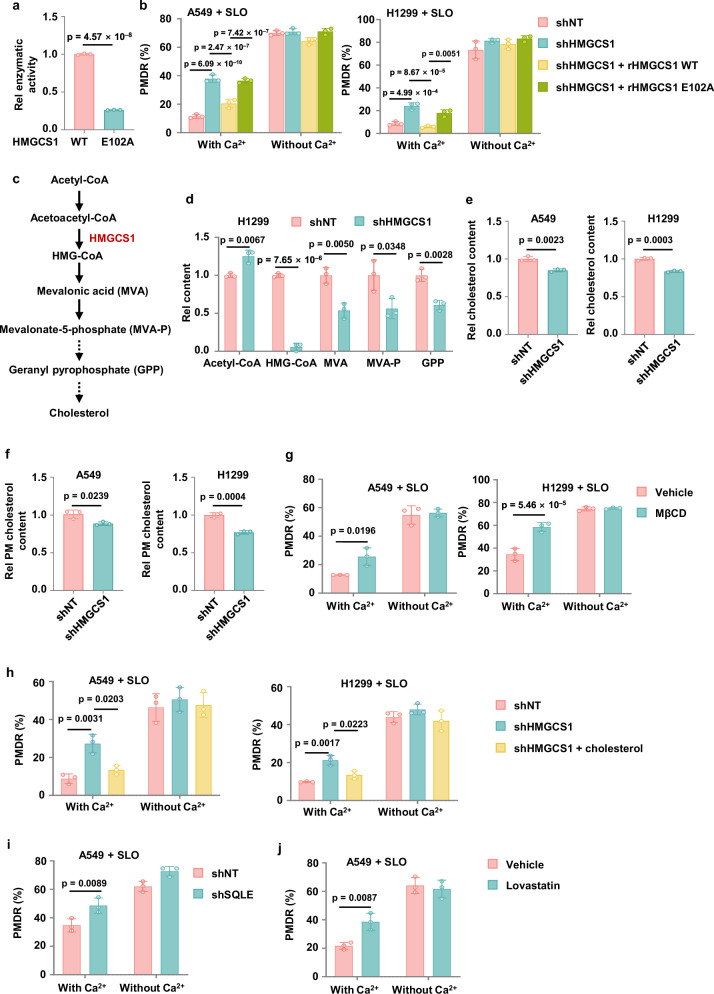
HMGCS1-dependent de novo cholesterol synthesis supports PM repair by producing cholesterol. **a** A549 cells stably expressing Flag-HMGCS1 WT or E102A were harvested and Flag-HMGCS1 proteins were immunoprecipitated and subjected to enzymatic activity assay. Rel, relative. **b** HMGCS1-depleted A549 or H1299 cells were rescued with rHMGCS1 WT or E102A. Cells were treated with or without SLO in PBS containing Ca²⁺ or in PBS free of Ca²⁺. **c** Schematic diagram of the mevalonic acid and cholesterol synthesis pathway. **d** HMGCS1-depleted H1299 cells were harvested, and the levels of metabolites shown in (**c**) were examined in these cells. **e**, **f** HMGCS1-depleted A549 or H1299 cells were harvested, and total cholesterol (**e**) and PM cholesterol (**f**) in these cells were examined. **g** Cholesterol was depleted using MβCD in A549 or H1299 cells. Cells were treated with or without SLO in PBS containing Ca²⁺ or in PBS free of Ca²⁺. **h** HMGCS1-depleted A549 or H1299 cells were rescued with MβCD-coated cholesterol. Cells were treated with or without SLO in PBS containing Ca²⁺ or in PBS free of Ca²⁺. **i** A549 cells were depleted of SQLE. Cells were treated with or without SLO in PBS containing Ca²⁺ or in PBS free of Ca²⁺. **j** A549 cells were treated with 10 μM Lovastatin for 12 h. Cells were treated with or without SLO in PBS containing Ca²⁺ or in PBS free of Ca²⁺. **a**, **b**, **d**–**j** Data represent the mean ± s.d. of three biologically independent experiments. **a**, **d**–**f** Two-tailed Student’s t-test. **b**, **h** Two-way ANOVA with Tukey’s multiple comparisons test. **g**, **i**, **j** Two-way ANOVA with Sidak’s multiple comparisons test. Also see Supplementary Fig. 2. Source data are provided as a Source Data file.

HMGCS1 acts as the first enzyme in the de novo cholesterol biosynthesis pathway, and cholesterol is a crucial component of PM. To determine whether HMGCS1-promoted PM repair depends on its enzymatic activity, we constructed an enzymatic-dead (ED) mutant (HMGCS1 E102A) of HMGCS1, which showed that HMGCS1 E102A had much lower activity than HMGCS1 WT (Fig. 2a). We then depleted endogenous HMGCS1 in A549 or H1299 cells with shRNA and rescued with rHMGCS1 WT or E102A. PI and Hoechst staining analyses revealed that rescued expression of rHMGCS1 WT, but not that of rHMGCS1 E102A, dramatically recovered PM integrity in HMGCS1-depleted cells after SLO treatment, while there was no discernible difference in PMDR between these groups of cells cultured without Ca^2+^ (Fig. 2b and Supplementary Fig. 2a), indicating that the catalytic activity of HMGCS1 is required for PM repair. Next, we measured the levels of the intermediates in the de novo cholesterol synthesis pathway (Fig. 2c). As shown in Fig. 2d, HMGCS1 depletion increased acetyl-CoA levels, while decreased levels of HMG-CoA, mevalonic acid (MVA), mevalonate-5-phosphate (MVA-P), and geranyl pyrophosphate (GPP) in A549 cells. Consistently, HMGCS1 depletion significantly decreased the total cholesterol levels as well as the PM cholesterol levels in A549 and H1299 cells (Fig. 2e, f).

To determine whether cholesterol is required for HMGCS1-promoted PM repair, we removed cholesterol in A549 or H1299 cells with methyl-β-cyclodextrin (MβCD) (Supplementary Fig. 2b). PI and Hoechst staining analyses showed that the elimination of cholesterol aggravated SLO-induced PM damage, while this difference disappeared when the cells were cultured in Ca^2+^-free PBS (Fig. 2g). Besides, we treated HMGCS1-depleted A549 or H1299 cells with MβCD-coated cholesterol to replenish cellular cholesterol and observed that the addition of cholesterol dramatically recovered PM integrity in HMGCS1-depleted cells after SLO treatment in Ca^2+^-containing PBS, while there was no difference in PMDR between these groups of the cells cultured in Ca^2+^-free PBS (Fig. 2h and Supplementary Fig. 2c). Furthermore, we inhibited de novo cholesterol synthesis by depleting squalene epoxidase (SQLE) with shRNA or inhibiting 3-hydroxy-3-methylglutaryl-coenzyme A reductase (HMGCR) with Lovastatin. PI and Hoechst staining analyses showed that SQLE depletion or HMGCR inhibition attenuated PM repair (Fig. 2i, j and Supplementary Fig. 2d). Furthermore, we generated a triple knockdown (KD) of HMGCS1, SQLE, and HMGCR to substantially suppress cholesterol synthesis in A549 cells. As shown in Supplementary Fig. 2e, compared to the KD of HMGCS1, the triple KD further aggravated SLO-induced PM damage. These results suggest that HMGCS1-dependent de novo cholesterol synthesis supports PM repair by providing cholesterol.

Intracellular cholesterol originates from extracellular uptake as well as de novo synthesis within cells, where it is then esterified and stored after undergoing intracellular esterification^21^. To determine whether esterification of cholesterol participates in PM repair, we inhibited the cholesterol esterification with acyl-coenzyme A:cholesterol acyltransferase (ACAT) inhibitor K604 or pyripyropene A (PPPA) in A549 cells and treated these cells with SLO. The inhibition of ACAT did not affect the levels of cholesterol in PM and SLO-induced PM damage (Supplementary Fig. 2f, g), suggesting that cholesterol esterification is dispensable for PM repair upon SLO treatment. Additionally, to assess whether extracellular uptake of cholesterol contributes to the PM repair, we compared SLO‑induced PMDR in HMGCS1‑depleted A549 cells cultured in medium containing either normal serum (NS) or lipoprotein‑deficient serum (LPDS, which is depleted of cholesterol and other lipids). As shown in Supplementary Fig. 2h, HMGCS1 knockdown resulted in a more pronounced increase in PMDR under LPDS conditions than under NS conditions, suggesting that extracellular uptake of cholesterol also supports the PM repair.

### Cholesterol supports PM repair by promoting PM localization of CHMP4B and providing building blocks

**Fig. 3:**
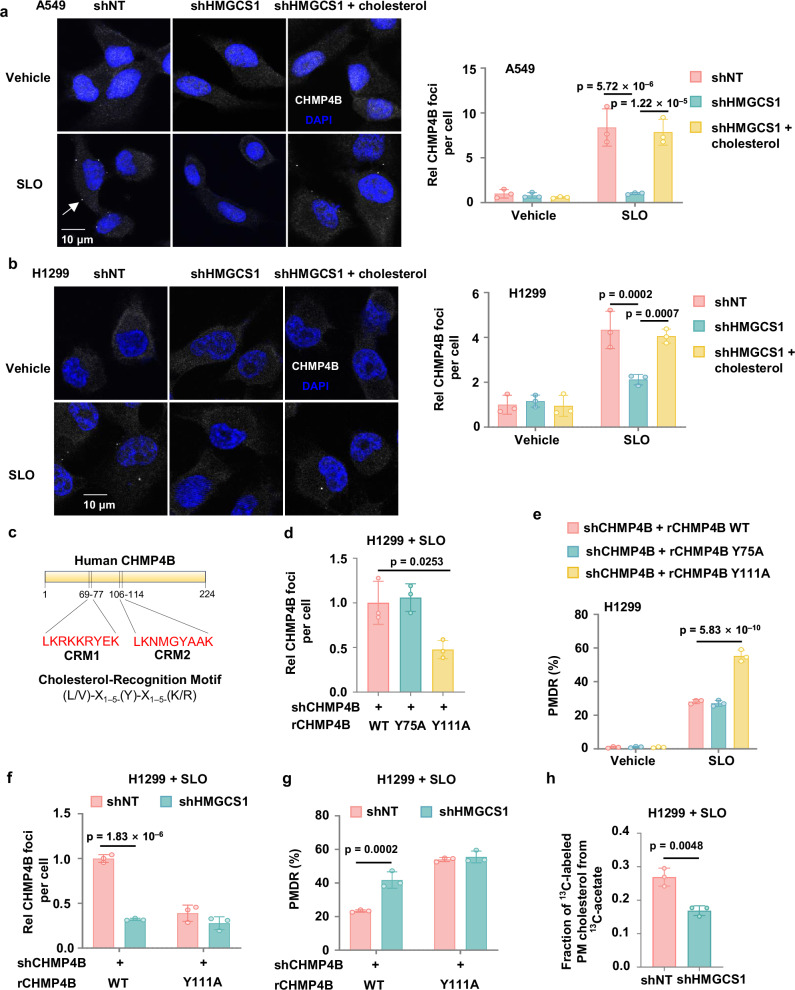
Cholesterol supports PM repair by promoting membrane localization of CHMP4B and providing building blocks. **a**, **b** HMGCS1-depleted A549 (**a**) or H1299 (**b**) cells were rescued with MβCD-coated cholesterol. IF staining was performed in these cells treated with or without SLO. Representative images (left) and statistical analysis (right) were shown. **c** Diagrammatic representation of the cholesterol-recognition motif in the CHMP4B protein. **d**, **e** CHMP4B-depleted H1299 cells were rescued with rCHMP4B WT, Y75A or Y111A. Cells were treated with SLO. IF was performed in these cells and relative CHMP4B foci per cell was calculated (**d**). PI and Hoechst staining were performed, and PMDR was calculated (**e**).** f**, **g** HMGCS1 was depleted in CHMP4B-depleted H1299 cells rescued with rCHMP4B WT or Y111A. Cells were treated with SLO. IF was performed in these cells and relative CHMP4B foci per cell was calculated (**f**). PI and Hoechst staining were performed, and PMDR was calculated (**g**). **h** HMGCS1 was depleted in H1299 cells. Cells were treated with ^13^C-actate for 72 h before treated with SLO. The conversion of ^13^C-acetate to PM cholesterol in these cells were measured. **a**, **b**, **d**–**h** Data represent the mean ± s.d. of three biologically independent experiments. **a**, **b**, **e** Two-way ANOVA with Tukey’s multiple comparisons test. **d** One-way ANOVA with Tukey’s multiple comparisons test. f,g. Two-way ANOVA with Sidak’s multiple comparisons test. **h** Two-tailed Student’s t-test. Also see Supplementary Fig. 3. Source data are provided as a Source Data file.

To investigate the mechanism underlying cholesterol-promoted PM repair, we replenished cholesterol in HMGCS1-depleted A549 or H1299 cells, followed by the treatment of SLO. Immunofluorescence (IF) staining and immunoblotting analyses showed HMGCS1 depletion dramatically decreased the membrane localization of CHMP4B, which was rescued by the addition of cholesterol (Fig. 3a, b and Supplementary Fig. 3a). In addition to exerting non-specific effects on membrane fluidity, cholesterol can specifically interact with membrane proteins, influencing both their structure and function via cholesterol-recognition motifs^22^. We thus analyzed the amino acid sequences of CHMP4B and identified two conserved cholesterol-recognition motifs (CRM) in CHMP4B, named CRM1 and CRM2, respectively (Fig. 3c). To specifically disrupt the interaction between cholesterol and CHMP4B, we generated CHMP4B mutants, including Y75A and Y111A, in which tyrosine (Y) was mutated into alanine (A). We then depleted CHMP4B in H1299 cells and rescued these cells with rCHMP4B WT, Y75A, or Y111A (Supplementary Fig. 3b). As depicted in Fig. 3d, compared to rCHMP4B WT, rCHMP4B Y111A, but not rCHMP4B Y75A, decreased membrane localization of CHMP4B in the cells after SLO treatment. Consistently, rCHMP4B Y111A aggravated PM damage as well compared to rCHMP4B WT (Fig. 3e). Moreover, we depleted HMGCS1 in CHMP4B-depleted H1299 cells rescued with rCHMP4B WT or rCHMP4B Y111A (Supplementary Fig. 3c). Immunofluorescence analysis revealed that HMGCS1 depletion reduced PM localization of CHMP4B and aggravated SLO-induced PM damage in cells rescued with rCHMP4B WT, while it failed to do so in cells rescued with rCHMP4B Y111A (Fig. 3f, g). Collectively, these results demonstrate that cholesterol binds to CHMP4B at CRM2 motif to increase its PM localization and promote PM repair.

To determine whether the PM localization of CHMP4B is necessary for cholesterol-supported PM repair, we performed sequence homology analysis based on a related study^23^ and identified phenylalanine at position 8 (F8) as a critical residue for the PM localization of human CHMP4B. We constructed an F8E mutation and confirmed that the mutation abrogated PM localization of CHMP4B under SLO treatment (Supplementary Fig. 3d). Next, we depleted endogenous CHMP4B in H1299 cells and rescued them with either rCHMP4B WT or F8E mutant (Supplementary Fig. 3e), followed by cholesterol pre‑treatment prior to SLO challenge. As shown in Supplementary Fig. 3f, cholesterol‑mediated enhancement of PM repair was evident in cells expressing rCHMP4B‑WT, but was abolished in cells expressing the rCHMP4B‑F8E mutant, suggesting that PM localization of CHMP4B is required for cholesterol-supported PM repair.

Besides, we depleted CHMP2B, another ESCRT component, in H1299 cells stably expressing either shNT or shHMGCS1. As shown in Supplementary Fig. 3g, under SLO treatment, HMGCS1 depletion impaired PM integrity, whereas cholesterol supplementation restored PM integrity in HMGCS1‑depleted cells expressing control siRNA (siNT). In contrast, this cholesterol‑mediated recovery was completely abolished in CHMP2B knockdown (siCHMP2B) cells (Supplementary Fig. 3g). The knockdown efficiency of CHMP2B siRNA was validated in Supplementary Fig. 3h. These results indicate that multiple ESCRT components, including CHMP2B, participate in the cholesterol-dependent PM repair process regulated by HMGCS1.

In addition, cholesterol is also an important component of PM. To confirm that HMGCS1-mediated de novo synthesis contributes to PM repair by providing building blocks, we conducted a ^13^C-labeled acetate tracing assay. As depicted in Fig. 3h, HMGCS1 depletion led to a decrease in acetate-derived PM cholesterol in the cells treated with SLO, suggesting that HMGCS1 also supports PM repair by supplying cholesterol for membrane repair as building blocks.

### HMGCS1-promoted PM repair prevents tumor cells from lymphocyte killing

**Fig. 4:**
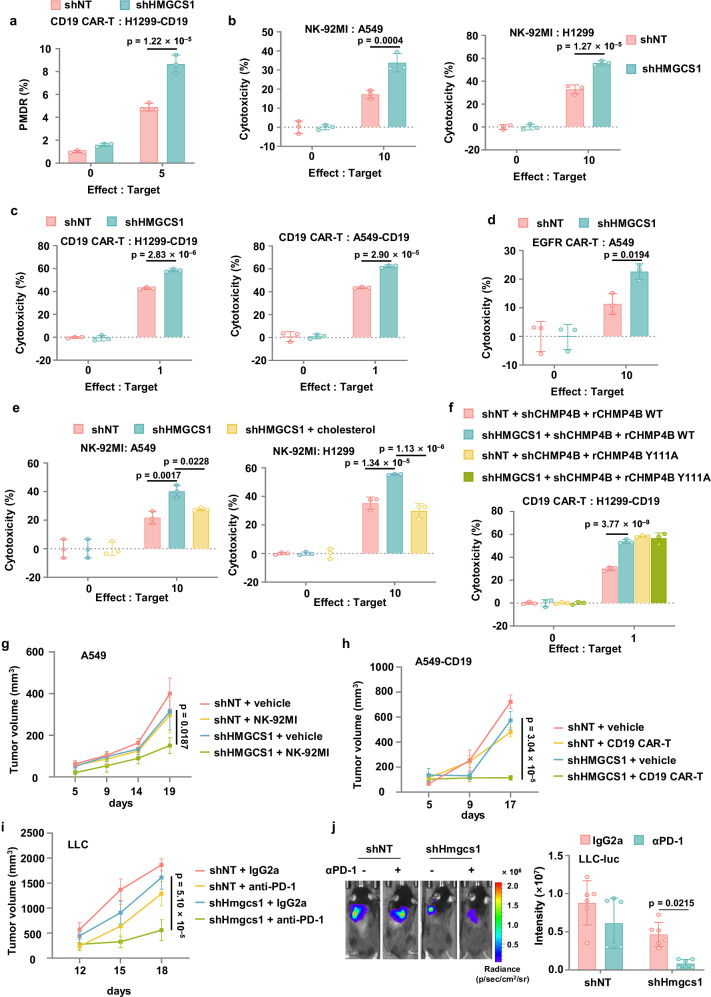
HMGCS1-promoted PM repair prevents tumor cells from lymphocyte killing. **a** HMGCS1 was depleted in H1299-CD19 cells. Cells were cocultured with CD19 CAR-T for 3 h. PI and Hoechst staining were performed, and PMDR of tumor cells was calculated. **b** HMGCS1 was depleted in A549 or H1299 cells. Cells were cocultured with NK-92MI for 4 h. **c** HMGCS1 was depleted in A549-CD19 or H1299-CD19 cells. Cells were cocultured with CD19 CAR-T for 24 h. **d** HMGCS1 was depleted in A549 or H1299 cells. Cells were cocultured with EGFR CAR-T for 24 h. **e** HMGCS1-depleted A549 or H1299 cells were rescued with MβCD-coated cholesterol. Cells were then cocultured with NK-92MI for 4 h. **f** HMGCS1 was depleted in CHMP4B-depleted H1299-CD19 cells and rescued with rCHMP4B WT or Y111A. Cells were cocultured with CD19 CAR-T for 24 h. **g** HMGCS1-depleted A549 cells were subcutaneously injected into female B-NDG mice. 5 days after inoculation, mice were treated with NK-92MI cells. Tumor growth over time was measured. **h** HMGCS1-depleted A549-CD19 cells were subcutaneously injected into female B-NDG mice. 5 days after inoculation, mice were treated with CD19 CAR-T cells. Tumor growth over time was measured. **i** Hmgcs1-depleted LLC cells were subcutaneously injected into female C57BL/6 mice (six mice per group). 5 days after inoculation, mice were treated with control IgG2a or anti-PD-1 antibody by intraperitoneal injection. Tumor growth over time was measured. **j** Hmgcs1-depleted LLC cells stably expressing luciferase (LLC-luc) were injected into the lungs of female C57BL/6 mice (six mice per group). 5 days after inoculation, mice were treated with control IgG2a or anti-PD-1 antibody by intraperitoneal injection. 13 days after inoculation, bioluminescence imaging was performed, and representative images of tumor growth were shown (left). **a**–**f** Data represent the mean ± s.d. of three biologically independent experiments. **g**–**j** Data represent mean ± s.d. from six mice. **a**–**d**, **j** Two-way ANOVA with Sidak’s multiple comparisons test. **e**–**i** Two-way ANOVA with Tukey’s multiple comparisons test. Also see Supplementary Figs. 4–6. Source data are provided as a Source Data file.

To confirm that HMGCS1 is required for the repair of CTLs-mediated PM damage, we depleted HMGCS1 in CD19-overexressed H1299 (H1299-CD19) cells and cocultured these cells with or without CD19 CAR-T cells. PI and Hoechst staining analyses showed that HMGCS1 depletion significantly increased PM damage in tumor cells under coculture conditions (Fig. 4a). Next, we cocultured HMGCS1-depleted A549 or H1299 cells with or without NK-92MI NK cells. HMGCS1 depletion markedly increased the sensitivity of lung cancer cells to NK cell-mediated killing (Fig. 4b). We also depleted HMGCS1 in H1299-CD19 or A549-CD19 cells and cocultured these cells with or without CD19 CAR-T cells. We found that HMGCS1 depletion enhanced CD19 CAR-T-induced tumor cell death (Fig. 4c). Similar results were obtained in the coculture system with A549 and EGFR CAR-T cells (Fig. 4d). In contrast, the overexpression of HMGCS1 has the opposite effect (Supplementary Fig. 4a). Notably, HMGCS1-dependent tumor cell resistance to NK-92MI killing was dependent on its enzymatic activity (Supplementary Fig. 4b). The pretreatment with cholesterol abrogated HMGCS1 depletion-enhanced tumor cell death after NK-92MI coculture (Fig. 4e). Additionally, we added the HMGCS1 inhibitor hymeglusin to co-culture systems of A549‑CD19 or H1299‑CD19 cells with CD19 CAR‑T cells. As shown in Supplementary Fig. 4c, hymeglusin treatment indeed enhanced CAR‑T cell‑mediated cytotoxicity against tumor cells, suggesting the translational potential of targeting HMGCS1 in combination with CAR‑T therapy.

Furthermore, while HMGCS1 depletion enhanced tumor cell sensitivity to CAR‑T killing in CHMP4B‑depleted H1299-CD19 cells rescued with rCHMP4B WT, this effect was lost in cells rescued with the rCHMP4B Y111A mutant (Fig. 4f). Additionally, we co‑cultured CHMP4B‑depleted H1299‑CD19 cells rescued with either rCHMP4B WT or the F8E mutant together with CD19 CAR‑T cells in the absence or presence of cholesterol. F8E mutation-mediated disruption of CHMP4B PM localization abrogated cholesterol‑mediated enhancement of tumor cell resistance to lymphocyte cytotoxicity (Supplementary Fig. 4d). Together, these results suggest that membrane-localized CHMP4B is essential for cholesterol-mediated promotion of tumor immune evasion.

HMGCS1 overexpression will produce geranylgeranyl diphosphate (GGPP) and farnesyl pyrophosphate (FPP), which can be used as geranylgeranylation and farnesylation substrates in protein prenylation^24^. To assess whether protein prenylation involved in ESCRT-mediated membrane repair, we treated A549 cells with the prenylation inhibitor L778123. As shown in Supplementary Fig. 4e, prenylation inhibition did not reverse the HMGCS1-mediated decrease in PMDR. We also pretreated HMGCS1-overexpressed A549-CD19 cells with the prenylation inhibitor L778123 prior to co‑culture with CD19 CAR‑T cells. The L778123 treatment did not diminish the enhanced resistance to lymphocyte killing conferred by HMGCS1 overexpression (Supplementary Fig. 4f). These results indicate that protein prenylation is not involved in HMGCS1-facilitated, ESCRT-mediated membrane repair.

Upon HMGCS1 depletion, levels of HMG‑CoA, MVA, MVA‑P, and GPP were reduced alongside cholesterol (Fig. 2d). To investigate whether the intermediate metabolites are involved in ESCRT-mediated membrane repair and tumor cell resistance to lymphocyte-mediated killing, we specifically increased the concentration of individual metabolites through approaches below. Specifically, to increase HMG-CoA levels, we overexpressed HMGCS1 while knocking down HMGCR. To elevate MVA, which is permeable to cells, we supplemented exogenous MVA while knocking down mevalonate kinase (MVK) to block its conversion to MVA-P. For MVA-P and GPP, which cannot enter cells directly, we supplemented MVA while knocking down phosphomevalonate kinase (PMVK) or farnesyl-diphosphate farnesyltransferase 1 (FDFT1), respectively, to allow intracellular accumulation of MVA-P or GPP through blocked downstream metabolism (Supplementary Fig. 5a–c). Importantly, none of these interventions—which effectively raised intracellular levels of HMG-CoA, MVA, MVA-P, or GPP—enhanced plasma membrane repair following SLO-induced damage, nor did they improve tumor cell resistance to lymphocyte-mediated killing (Supplementary Fig. 5d, e). Of note, the levels of cholesterol were not altered by these interventions (Supplementary Fig. 5f), which may be due to the addition of upstream metabolites, together with the possibly incomplete knockdown of downstream enzymes. Together, these results suggest HMGCS1 confers tumor cells resistance to immune killing specifically through cholesterol-regulated CHMP4B.

Next, we subcutaneously implanted A549 cells stably expressing shNT or shHMGCS1 into B-NDG immunodeficient mice. After the mice developed palpable tumors, NK-92MI cells were injected intravenously into these mice. As shown in Fig. 4g and Supplementary Fig. 6a, the depletion of HMGCS1 dramatically enhanced the efficacy of NK therapy. Similarly, we subcutaneously implanted A549-CD19 cells expressing shNT or shHMGCS1 into B-NDG immunodeficient mice and treated these mice with or without CD19 CAR-T cells. The depletion of HMGCS1 elevated the efficacy of CAR-T therapy (Fig. 4h and Supplementary Fig. 6b). Importantly, the combination of HMGCS1 knockdown with either CAR-T or NK cell treatment significantly extended overall survival compared to HMGCS1 depletion alone (Supplementary Fig. 6c, d).

Furthermore, we constructed Hmgcs1-depleted mouse Lewis lung cancer (LLC) cells and implanted these cells subcutaneously into c57BL/6 mice, followed by anti-PD-1 treatment. As shown in Fig. 4i, anti-PD-1 treatment slightly dampened tumor growth in shNT group, while in shHmgcs1 group, anti-PD-1 treatment dramatically dampened tumor growth. Additionally, we established an orthotopic lung cancer model by implanting luciferase-expressing LLC (LLC-luc) cells, with or without Hmgcs1 knockdown, into the left lung of C57BL/6 mice. The mice were subsequently administered either control IgG2a or anti‑PD‑1. Tumor progression and mouse survival were then assessed. As shown in Fig. 4j, Hmgcs1 knockdown slightly attenuated tumor growth in the control IgG2a-treated group and significantly potentiated the efficacy of anti‑PD‑1 therapy. Importantly, the combination of Hmgcs1 knockdown and anti‑PD‑1 treatment produced the most substantial survival benefit (Supplementary Fig. 6e).

Collectively, these results demonstrate that HMGCS1-dependent PM repair shields tumor cells against immune attack, and that targeting HMGCS1 improves the efficacy of anti-PD-1 immunotherapy.

### HMGCS1 expression is upregulated by c-Jun

**Fig. 5:**
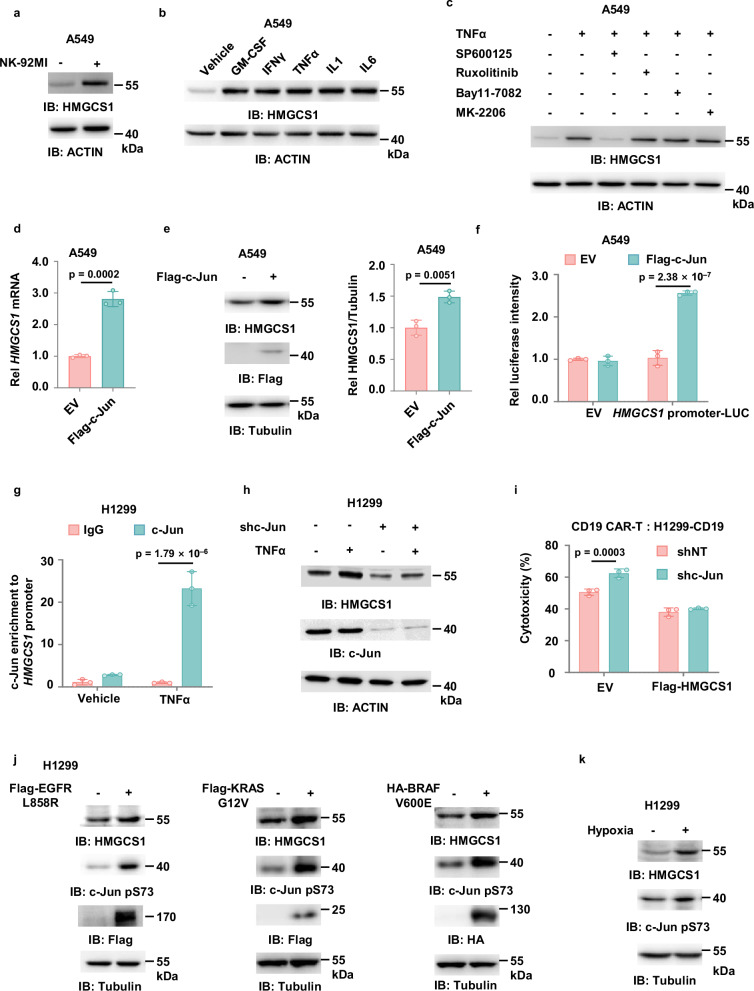
HMGCS1 expression is upregulated by c-Jun. **a** A549 cells were cocultured with NK-92MI cells for 12 h. A549 cells were harvested and subjected to immunoblotting analyses. **b** A549 cells were treated with 20 ng/ml GM-CSF, IFNγ, TNFα, IL1, or IL6 for 12 h. Cells were harvested and subjected to immunoblotting analyses. **c** A549 cells were pre-treated with 10 μM SP600125, Ruxolitinib, Bay11-7082, or MK-2206 for 12 h, followed by treatment with 20 ng/ml TNFα for an additional 12 h. **d** A549 cells stably expressing EV or Flag-c-Jun were harvested, and the mRNA level of *HMGCS1* in these cells was examined. **e** A549 cells expressing EV or Flag-c-Jun were harvested and the protein level of *HMGCS1* in these cells was examined (left). Semi-quantitative scoring was carried out (right). **f** The luciferase reporter construct containing *HMGCS1* promoter was cotransfected with EV or Flag-c-Jun into A549 cells. The relative levels of luciferase activity of Firefly were normalized to the levels of luciferase activity of control Renilla. **g** H1299 cells were treated with or without TNFα. Cell were harvested and subjected to CHIP assay. **h** c-Jun-depleted H1299 cells were treated with or without TNFα. Cells were harvested and subjected to immunoblotting analyses. **i** c-Jun-depleted H1299-CD19 cells were overexpressed with EV or Flag-HMGCS1. Cells were cocultured with CD19 CAR-T for 24 h.** j** H1299 cells were transfected with EV, Flag-EGFR L858R, Flag-KRAS G12V, or HA-BRAF V600E. Cells were harvested and subjected to immunoblotting analyses. **k** H1299 cells were treated with 200 μM CoCl_2_ for 24 h. Cells were harvested and subjected to immunoblotting analyses. **a**–**c**, **e**, **h**, **j**, **k** Immunoblots are representative of three biologically independent experiments. **d**–**g**, **i** Data represent the mean ± s.d. of three biologically independent experiments. **d**, **e** Two-tailed Student’s t-test. **f**, **g**, **i** Two-way ANOVA with Sidak’s multiple comparisons test. Also see Supplementary Fig. 7. Source data are provided as a Source Data file.

To investigate how HMGCS1 expression is regulated, we cocultured NK-92MI cells with A549 or H1299 cells. Immunoblotting analysis showed that NK-92MI cells coculture induced the upregulation of HMGCS1 expression in tumor cells (Fig. 5a and Supplementary Fig. 7a). Immune cells secrete cytokines to mediate their immune regulatory functions. To identify the cytokine that induces the upregulation of HMGCS1 expression, we treated A549 cells with several cytokines reported to be present in the tumor microenvironment (TME) and discovered that the stimulation of many cytokines, including TNFα, GM-CSF, IFNγ, IL1, and IL6, upregulated the expression of HMGCS1 in tumor cells (Fig. 5b and Supplementary Fig. 7b).

To explore the mechanism of how cytokines regulate the expression of HMGCS1, we treated A549 or H1299 cells with the inhibitors of TNFα-related signaling pathways, including c-Jun N-terminal kinase (JNK), Janus kinase (JAK), nuclear factor kappa B (NF-κB) and phosphatidylinositol-3 kinase (PI3K)-AKT, and found that the pathway inhibitor JNK inhibitor SP600125 significantly inhibited the upregulation of HMGCS1 expression in lung cancer cells after TNFα stimulation (Fig. 5c and Supplementary Fig. 7c). Upon activation, JNK translocates to the nucleus and activates transcription factors such as c-Jun, which regulates the downstream gene transcription. We then overexpressed c-Jun in A549 or H1299 cells. Real-time PCR and immunoblotting analyses showed that c-Jun overexpression dramatically increased the expression of HMGCS1 (Fig. 5d, e, and Supplementary Fig. 7d, e). Besides, we constructed a luciferase reporter system containing *HMGCS1* promoter and observed that c-Jun overexpression increased the luciferase activity of *HMGCS1* promoter (Fig. 5f). Chromatin immunoprecipitation (ChIP) assays showed that c-Jun was recruited to *HMGCS1* promoter in tumor cells after TNFα treatment (Fig. 5g). Additionally, the depletion of c-Jun in A549 or H1299 cells abolished the upregulation of HMGCS1 expression induced by TNFα (Fig. 5h and Supplementary Fig. 7f). Consistently, the depletion of c-Jun in H1299 cells increased the sensitivity of tumor cells to CAR-T-mediated immune cell killing, which was reduced by HMGCS1 overexpression (Fig. 5i and Supplementary Fig. 7g). Finally, to evaluate the role of c‑Jun in tumor cell resistance to immune‑mediated killing, we overexpressed or depleted c‑Jun in A549‑CD19 and H1299‑CD19 cells, followed by co‑culture with CD19 CAR‑T cells. Overexpression of c‑Jun enhanced tumor cell resistance to CAR‑T killing, whereas c‑Jun depletion sensitized tumor cells to lymphocyte‑mediated cytotoxicity (Supplementary Fig. 7h, i).

c-Jun operates within multiple layers of a complex regulatory network, where it can cross-talk, amplify, and integrate various signals involved in tissue development and disease^25^. This suggests that multiple signals may promote tumor immune escape through c-Jun-regulated HMGCS1. To test this hypothesis, we overexpressed the well-known oncogenic mutations that activate c-Jun, such as EGFR L858R, KRAS G12V, and BRAF V600E, in H1299 cells. We found that EGFR L858R, BRAF V600E, or KRAS G12V overexpression indeed promoted HMGCS1 expression (Fig. 5j). In addition to oncogenic mutations, we found that hypoxia also activated c-Jun and increased HMGCS1 expression (Fig. 5k). These results highlight the widespread regulation of c-Jun-mediated upregulation of HMGCS1 to enhance tumor immune escape.

Taken together, these results demonstrate that oncogenic activation, cytokine signaling, and hypoxia activate c-Jun to upregulate HMGCS1 expression in lung cancer cells, thereby enhancing tumor cell resistance to immune cell killing.

### HMGCS1 expression is associated with immunotherapy efficacy

**Fig. 6:**
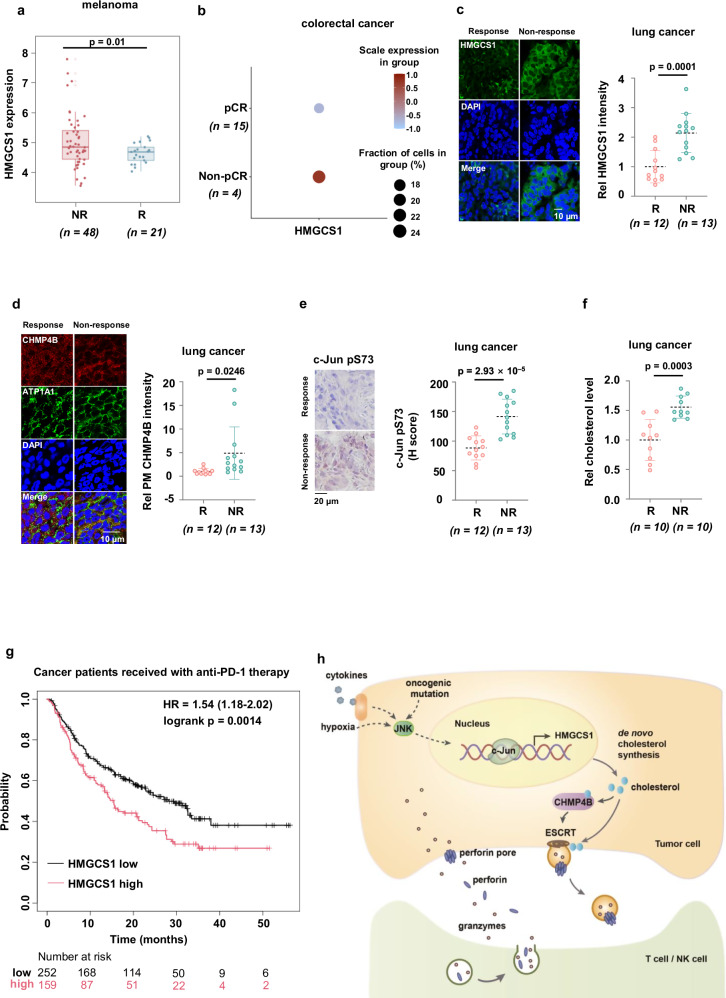
HMGCS1 expression is associated with immunotherapy efficacy. **a** GEO RNA-sequencing data from melanoma patients were analyzed. *HMGCS1* mRNA levels in tumor tissues were compared between patients who responded or did not respond to immunotherapy (two-side wilcoxon signed-rank test). The boxes represent the median and the first and third quartiles, while the whiskers indicate the minimum and maximum values of all data points. **b** Dot plot of HMGCS1 expression in colorectal tumors from individuals receiving with immunotherapy. Mean expression is shown as color and is standard scaled (binarized), whereas dot size represents the fraction of samples with expression. pCR, pathological complete response. (pCR, *n*  =  15; Non-pCR, *n* = 4). **c** IF staining was performed using anti-HMGCS1 antibody on tumor tissues from lung cancer patients after ICB treatment (Response *n*  =  12; Non-response *n*  =  13). Representative images of IF staining were shown (left). The average intensity of HMGCS1 in tumor tissues was calculated (right, two-tailed Student’s t-test). **d** IF staining was performed using anti-CHMP4B antibody and anti-ATP1A1 (indicating PM) antibody on tumor tissues from lung cancer patients after ICB treatment (Response *n*  =  12; Non-response *n*  =  13). Representative images of IF staining were shown (left). The average intensity of PM CHMP4B in tumor tissues was calculated (right, two-tailed Student’s t-test). **e** IHC staining was performed using anti-phospho-c-Jun S73 antibody on tumor tissues from lung cancer patients after ICB treatment (Response *n*  =  12; Non-response *n*  =  13). Representative images of IHC staining were shown (left). Semiquantitative scoring of phospho-c-Jun (using a scale from 0 to 300) in tumor tissues was carried out (right, two-tailed Student’s t-test). **f** Cholesterol content in tumor tissues from lung cancer patients after ICB treatment (Response *n*  =  10; Non-response *n*  =  10) was measured. Two-tailed Student’s t-test. **g** Overall survival duration of cancer patients receiving with immunotherapy with high or low HMGCS1 expression (high HMGCS1 *n*  =  325; low HMGCS1 *n*  =  599) was compared using the online tool (https://www.kmplot.com/analysis/index.php?p=service&cancer=immunotherapy). Logrank test was performed. HR, Hazard ratio. **h** Schematic model of HMGCS1-dependent cholesterol biosynthesis promotes tumor immune evasion by facilitating PM repair. **c**–**f** Data are shown as the mean ± s.d. of tumors from lung cancer patients. Also see Supplementary Fig. 8. Source data are provided as a Source Data file.

To define the relationship between HMGCS1 expression and immunotherapy efficacy, we used data from the GEO database, which includes RNA-seq expression profiles and annotations related to immunotherapy efficacy. In melanoma data, we divided patient expression profiles into two groups based on the efficacy of anti-PD-1 therapy (GSE91061). As shown in Fig. 6a, the patients who exhibited no response to immunotherapy had higher HMGCS1 expression in tumor tissues. We also analyzed the single-cell sequencing data from colorectal cancer (CRC) patients receiving with immunotherapy^26^ and found that higher HMGCS1 expression was also observed in the tumor tissues from CRC patients who did not respond to immunotherapy (Fig. 6b). Of note, we confirmed the role of HMGCS1 in PM repair in A375 melanoma cells and HCT116 colorectal cancer cells by depleting HMGCS1 in both cells. Similarly, HMGCS1 depletion greatly sensitized both cell types to SLO-induced PM damage (Supplementary Fig. 8a, b).

Moreover, we performed IF analysis with anti-HMGCS1, anti-CHMP4B, and anti-ATP1A1 (plasma membrane marker) antibodies and immunohistochemistry (IHC) analysis with anti-phospho-c-Jun S73 antibody in tumor tissues from lung cancer patients receiving anti-PD-1-based immunotherapy. We observed that non‑responders exhibited significantly higher levels of HMGCS1, PM CHMP4B, and phospho‑c‑Jun in their tumor tissues compared to responders (Fig. 6c–e). Additionally, tumor cholesterol levels were also elevated in non‑responding patients (Fig. 6f). Moreover, we compared overall survival duration of cancer patients receiving with immunotherapy with high or low HMGCS1 expression using the online tool (https://www.kmplot.com/analysis/index.php?p=service&cancer=immunotherapy)^27,28^, and observed that higher HMGCS1 expression correlated with worse prognosis in cancer patients receiving with immunotherapy (Fig. 6g). Collectively, these results suggest the potential of HMGCS1 expression as a biomarker to predict the efficacy of immunotherapies.

## Discussion

Cytotoxic lymphocytes kill tumor cells by releasing perforin, a pore-forming protein that disrupts the PM of tumor cells, creating channels. These channels facilitate the entry of granzymes and other bioactive molecules, leading to tumor cell death^8^. A recent study demonstrates that ESCRT complex-dependent membrane repair system enhances tumor cell resistance to immune killing by preventing perforin-mediated pore formation in PM^7^. Meanwhile, Stefani et al. reported endolysosomal protein LITAF protects against pore-forming protein-induced cell death by promoting membrane repair at endosomal membranes, leading to the expulsion of damaged membranes as exosomes^29^. However, it is still unclear whether and how tumor cells undergo metabolic reprogramming to repair perforin-induced PM damage, thereby aiding their resistance to lymphocyte-mediated killing. In this study, we demonstrate a unique mechanism of HMGCS1-promoted PM repair by producing cholesterol, which not only functions a signal molecule to promote ESCRT-mediated membrane repair process, but also acts as the building blocks to supply membrane repair. Moreover, we establish the critical role of HMGCS1-promoted PM repair in tumor cell resistance to lymphocytes-mediated cytotoxicity and ultimately PD-1 inhibitors, CAR-T or NK cells-mediated immunotherapy resistance (Fig. 6h), implicating the therapeutic potential of targeting HMGCS1 to improve the efficacy of immunotherapies.

Cholesterol serves as a vital metabolite essential for the biological functions of mammalian cells. Its concentration, whether at the cellular or systemic level, is associated with numerous diseases, including obesity, heart disease, and cancer. Several groups have revealed the relationship between hypercholesterolemia and heightened cancer risk using clinical data. In addition to systemic cholesterol, dysregulated cellular cholesterol, resulting from heightened biosynthesis or uptake, drives the malignant characteristics of cancer cells^30–32^. Besides acting as an important membrane constituent required for rapid proliferation of tumor cells, cholesterol can also modulate signaling pathways involved in tumorigenesis and cancer progression by covalently modifying proteins including hedgehog and smoothened^33,34^. Cholesterol can facilitate the formation of specialized membrane microdomains to inhibit the transforming ability of Src family kinases and activate canonical Wnt signaling as well^35,36^. Furthermore, cholesterol and cholesterol-derived metabolites can induce CD8^+^ T cell exhaustion and dampen DC cell function in the tumor microenvironment^37,38^. Mahmut et al. found that cholesterol depletion leads to the stiffening of cancer cells, which in turn enhances T cell forces, thereby improving the effectiveness of adoptive T cell immunotherapy^39^. In contrast, in our study, we reveal a unique role of cholesterol in immune cell killing-induced PM repair in tumor cells. Furthermore, we uncover that two mechanisms mediate such cholesterol-promoted PM repair. On one hand, cholesterol acts as a building block to supply membrane repair. On the other hand, cholesterol binds to CHMP4B, an important constituent of ESCRT complex, to facilitate its PM localization to promote PM repair and enhance tumor cell resistance to lymphocyte-mediated cytotoxicity. In addition, we performed a systematic in silico analysis to examine other core ESCRT proteins for the presence of established cholesterol-recognition motifs ((L/V)-X₁–₅-(Y)-X₁–₅-(K/R)). Notably, our analysis indicated that many of these proteins also contain putative cholesterol-recognition motifs (Supplementary Fig. 9), suggesting that cholesterol-dependent regulation of the ESCRT machinery may not be limited to CHMP4B, and that targeting these interactions in other ESCRT components could represent a promising direction for future therapeutic development.

Cellular cholesterol levels are regulated by the balance between de novo biosynthesis, uptake, export, and storage. Cholesterol is synthesized from acetyl-CoA through approximately 30 reactions, with HMGCR and squalene monooxygenase serving as the rate-limiting enzymes. It can also be absorbed from the blood via low-density lipoprotein (LDL) receptors on polarized cells. Excess cholesterol is exported by ABCA1, ABCG1, ABCG5/ABCG8, or converted to cholesteryl ester (CE) by ACAT for storage or secretion in lipoproteins^21^. In our study, we found that, in contrast to the inhibition of esterification, which had no effect on the levels of PM cholesterol in A549 cells (Supplementary Fig. 2f), the inhibition of de novo cholesterol biosynthesis dramatically reduces PM cholesterol levels, exacerbates SLO-induced PM damage and sensitizes tumor cells to lymphocyte killing (Figs. 1c, 2i, 2j, Supplementary Fig. 2f and Fig. 4), highlighting the therapeutic potential of statins to improve immunotherapy efficacy. In addition, cells cultured under LPDS conditions exhibited elevated PMDR (Supplementary Fig. 2h), indicating that extracellular cholesterol uptake may contribute to PM repair. This observation suggests that reducing dietary cholesterol intake could enhance the efficacy of immunotherapy.

Notably, although inhibiting both HMGCS1 and HMGCR impairs PM repair (Figs. 1c and 2j), we propose that targeting HMGCS1 may offer a wider therapeutic window. This is because HMGCS1 plays a non‑rate‑limiting role in cholesterol synthesis and is specifically upregulated during tumor immune evasion. In contrast, HMGCR serves as the classic rate‑limiting enzyme of the mevalonate pathway, whose activity is tightly regulated to maintain cholesterol homeostasis in both normal and tumor cells. Consequently, inhibiting HMGCR is likely to cause broad metabolic disturbances across cell types, which could result in significant systemic side effects.

Given the critical role of the de novo cholesterol biosynthesis in tumor PM repair and immune evasion, we further investigated its regulation in lung cancer. We found that extrinsic factors from the tumor microenvironment, such as cytokines and hypoxia, as well as intrinsic oncogenic activation, activate JNK signaling to upregulate HMGCS1 expression in tumor cells, which enhances the tumor cells’ resistance to lymphocyte killing. Our finding not only elucidates the mechanism by which extrinsic and intrinsic factors regulate cholesterol metabolism in tumor cells, but also highlights the therapeutic potential of targeting these signaling pathways to disrupt tumor immune escape.

## Methods

### Ethics statement

The use of human lung tumor specimens and associated data was approved by the Institutional Review Board of Shanghai Chest Hospital, and the study was performed in accordance with the approved protocol. Informed consent was obtained from all patients.

All animal experimental protocols were approved by the Institutional Animal Care and Use Committee (IACUC) of the Shanghai Institute of Biochemistry and Cell Biology and complied with all relevant ethical regulations. Throughout the study, no tumor exceeded the IACUC-permitted maximum size of 1.5 cm in diameter.

No sex and gender-based analysis was performed. The study examined the expression of genes of interest in tumor tissues from lung cancer patients undergoing anti-PD-1-based immunotherapy, a molecular event independent of patient sex or gender.

### Mice

Six-week-old female B-NDG mice were purchased from Biocytogen. Six-week-old female C57BL/6 mice were purchased from SLAC (Shanghai, China). Littermates were randomly assigned to experimental groups. Sex was not considered a biological variable in the study design. All experiments were conducted with female mice to limit complications of male territorial behavior and fighting during long-term cancer experiments. Mice were housed under specific pathogen-free conditions with a standard chow diet and maintained at a room temperature of 20–26 °C with 40-70% humidity on a 12-h light/12-h dark cycle.

### Materials

#### Antibodies

Primary antibodies were used against: HMGCS1 (17643-1-AP, Proteintech Group); Flag (20543-1-AP, Proteintech Group); Actin (60008-1-lg, Proteintech Group); Tubulin (T5201, Sigma-Aldrich); SQLE (12544-1-AP, Proteintech Group); CHMP4B (13683-1-AP, Proteintech Group); CHMP4B (68811-2-Ig, Proteintech Group); ATP1A1 (14418-1-AP, Proteintech Group); CDH1 (20874-1-AP, Proteintech Group); c-Jun (9165 T, Cell Signaling Technology); c-Jun pS73 (3270 T, Cell Signaling Technology); HMGCR (ab174830, Abcam). The following secondary antibodies were used: goat-anti-mouse IgG second antibody (31160, Thermo); goat-anti-rabbit IgG second antibody (31210, Thermo); goat-anti-mouse cy3 (115-165-146, Jackson ImmunoResearch); goat-anti-rabbit Alexa Fluor 488 (A11034, Thermo). The primary antibodies were used at a 1:1000 dilution for immunoblotting and a 1:300 dilution for IF. Secondary antibodies were used at 1:3000 dilution for immunoblotting and a 1:200 dilution for IF.

#### Reagents

Human IL1, IL6, GM-CSF, IFNγ and TNFα were brought from Peprotech. JNK inhibitor (SP600125), JAK1/2 inhibitor (Ruxolitinib), NF-κB inhibitor (BAY 11-7082) and AKT inhibitor (MK-2206) were obtained from Selleckchem. L778123 was brought from MedChemExpress. Hymeglusin was brought from Targetmol. SLO was brought from Beyotime Biotechnology. PI and Hoechst 33342 were brought from Invitrogen. Cholesterol and MβCD were brought from Sigma-Aldrich. Amplex Red Cholesterol Assay Kit and EZ-Link™ Sulfo-NHS-SS-Biotin were brought from Thermo. ^13^C-acetate was brought from Cambridge Isotope Laboratories.

### DNA constructs and mutagenesis

PCR-amplified human HMGCS1, CHMP4B, c-Jun, EGFR, KRAS, and BRAF were cloned into pCDH-Flag, pFlag-CMV, or pcDNA3.0-HA vector. Mutations were constructed using the QuikChange site-directed mutagenesis kit (Stratagene).

The pGIPZ shNT was generated with the control oligonucleotide 5′-GCTTCTAACACCGGAGGTCTT −3′. pGIPZ human *HMGCS1* shRNA was generated with 5′- GAACAGAGACAATCATCGA −3′ oligonucleotide. pGIPZ human *CHMP4B* shRNA was generated with 5′- GAAGAGATGTTAAGCAAGA −3′ oligonucleotide. pGIPZ human *c-Jun* shRNA was generated with 5′- GGACCTTATGGCTACAGTA −3′ oligonucleotide. pGIPZ human *SQLE* shRNA was generated with 5′- TCAGGCTCTTTATGAATTA −3′ oligonucleotide. pTRIPZ human *HMGCR* shRNA was generated with 5′- ACAGAATGTTGGTAGTTCA −3′ oligonucleotide. The siRNA oligonucleotides to human *CHMP2B*, *MVK*, *PMVK* and *FDFT1* were purchased from GenePharma, and the sequences are as follows: siNT, 5’- UUCUCCGAACGUGUCACGUTT −3’; siCHMP2B, 5’-UCGAGCAGCUUUAGAGAAATT −3’; siMVK, 5’- GGGAAGAUUUCAUCCUUAATT −3’; siPMVK, 5’- CCAUCUGGCUGGUGAGUGATT −3’; siFDFT, 5’-UUGCUAAGCCGGAGAAUAUTT −3’.

### Cell culture

The human lung cancer cell lines H1299 and A549, the mouse Lewis lung cancer cell line (LLC), the human melanoma cell line A375, the human colorectal cancer cell line HCT116, and the HEK293T cell line were kindly provided by the Type Culture Collection of the Chinese Academy of Sciences. Cells were maintained in Dulbecco’s modified Eagle’s medium (DMEM) supplemented with 10% fetal bovine serum (FBS) and antibiotics. NK-92MI cells were brought from Wuhan Pricella Biotechnology and maintained in Minimum Essential Medium α (MEMα) supplemented with 12.5% FBS, 12.5% horse serum, 0.1% β-mercaptoethanol, 0.2 mM inositol, 0.2 mM folic acid, and antibiotics. Cells were incubated in 5% CO_2_ at 37 °C. All cell lines were authenticated using the short tandem repeat (STR) method and were tested negative for mycoplasma.

### Cell viability assay

A totatl of 2 × 10^5^ A549 or H1299 cells were plated in 12-well plate and cultured for 24 h. Cell viability was assessed using trypan blue staining.

### Cell proliferation assay

1000 cells per well were plated in a 96-well plate and cultured for the indicated days. Cells were fixed with trichloroacetic acid and stained with SRB solution, followed by rinsing the plates four times with 1% acetic acid and solubilizing the protein-bound dye with 10 mM Tris base solution. The absorbance was measured at 560 nm. Relative cell proliferation was assessed by the OD560 values and normalized to day 1.

### siRNA screening

We selected 111 human key metabolic enzymes from the Enzymes Database^40^. A complete list of the genes encoding these enzymes is provided in Supplementary Table 1. All siRNAs targeting the genes were obtained from Dharmacon (Thermo). A549 cells were plated at 500 cells per well in black-walled 384-well plates (Nunc), and siRNA transfection was performed robotically using the Multidrop Combi (Thermo). 72 hours after transfection, cells were stained with Hoechst for 20 min, followed by incubation with SLO and PI for 5 min. The cells were then imaged under 4 × magnification, and PI-positive cell counts, as well as total cell counts, were determined using the Ensight high-throughput screening system. The ratio of PI-positive cells to total cells was defined as the plasma membrane damage rate (PMDR).

To ensure statistical robustness, the screen was designed with four independent siRNAs per gene, each performed in triplicate. A gene was considered a candidate if its knockdown increased the PMDR by >1.5-fold compared to the control (*p* < 0.05). HMGCS1 emerged as the top hit because it was the only candidate for which all four independent siRNAs significantly increased PMDR in A549 cells—representing the highest number of effective siRNAs across the entire screen. This consistent and robust phenotype formed the primary rationale for selecting HMGCS1 as the focus of subsequent investigation. The full siRNA screening result can be found in Supplementary Table 2.

### Immunoblotting analysis

Cells were lysed in cold RIPA buffer supplemented with 1 × protease inhibitor cocktail on ice for 30 min. After centrifugation (14,000 × *g*, 10 min, 4 °C), protein extracts were denatured in loading buffer at 95 °C for 10 min and subjected to SDS-PAGE. Separated proteins were transferred to nitrocellulose membranes, which were then probed with specific primary antibodies at 4 °C overnight, followed by incubation with HRP-conjugated secondary antibodies for 1 h at room temperature. After washing with TBST, immunoreactive bands were visualized using enhanced chemiluminescence (ECL).

### Immunofluorescence staining and semi-quantification analyses

For immunofluorescence staining on tumor cells, cells were fixed and incubated with primary antibodies, followed by Alexa Fluor dye-conjugated secondary antibodies and DAPI, according to standard protocols. Cell imaging was performed using a Leica TCS SP8 WLL confocal laser scanning microscope (Leica). Three fields per section were randomly selected and captured at 630 × magnification. The number of tumor cells and CHMP4B foci was then counted.

For immunofluorescence staining on human lung cancer tissues, tissues were fixed and incubated with primary antibodies, Alexa Fluor dye-conjugated secondary antibodies, and DAPI according to standard protocols. Five fields per section were randomly selected and captured at 630 × magnification. HMGCS1 intensity and PM CHMP4B intensity were calculated with ImageJ.

### Immunohistochemistry

The tissue sections from paraffin-embedded human lung cancer tissues were stained with antibodies as indicated. We quantitatively scored the tissue sections according to the percentage of positive cells and staining intensity. We rated staining intensity on a scale of 0 to 3: 0, negative; 1, weak; 2, moderate; 3, strong. We assigned the following proportion scores: X indicates the percentage of the tumor cells that were stained (0 ≤ [X1 + X2 + X3] ≤ 100), where X1 indicates weak staining, X2 moderate staining, and X3 strong staining. The score (H score) was obtained using the formula: 1 × X1 + 2 × X2 + 3 × X3, giving a range of 0 to 300.

### Quantitative realtime PCR

Total RNA was extracted with Trizol reagent (Life Technologies). cDNA was prepared by a HiScript II Q RT SuperMix for qPCR Kit (Vazyme). Quantitative realtime PCR analysis was performed using a Roche LightCycler96. Data were normalized to the expression of a control gene (β-actin) for each experiment.

### Luciferase reporter assay

A549 cells were cotransfected with pGL4-*HMGCS1* promoter reporter, control Renilla, and c-Jun plasmids. 24 h after transfection, cells were harvested for the measurement of luciferase activity. Luciferase activities were measured using a dual luciferase reporter assay kit (Promega) according to the manufacturer’s instructions.

### Chromatin immunoprecipitation

ChIP assays were performed as previously described^41^. The rabbit monoclonal antibody against c-Jun was used in ChIP assays with the rabbit monoclonal IgG serving as a negative control. The enrichment of c-Jun to HMGCS1 promoter was assessed by quantitative realtime PCR. 1% of sonicated DNA before addition of antibodies was used as the input. The primers used for realtime PCR were: CATTCTCATGACCGTCTGGCT (forward); TCCATTCCACAGTATTGCCCA (reverse).

### HMGCS1 activity assay

HMGCS1 activity was measured in 96-well plates at room temperature by monitoring the production of CoASH. The change in absorbance at 412 nm was measured using BioTek Synery Neo Multi-Mode Plate Reader (BioTek). Assay was performed in HMGCS1 reaction buffer containing 67 mM Tris-HCl (pH 8.0), 130 μM DTNB, 130 μM Ac-CoA, 7 μM acetoacetyl-CoA. HMGCS1 was added to start the reaction.

### Metabolites measurements

For metabolite measurements, all solutions, including PBS and 80% methanol: 20% water, were degassed by bubbling with nitrogen to remove oxygen. After the cell culture media were quickly aspirated, cells were rinsed with ice-cold PBS. After the cells were transferred to dry ice, 1 ml of dry ice cold solution of 80% methanol: 20% water (spiked with 20 μM 5-Fluorouracil, Sigma, 51-21-8) was added and quickly spread around by tilting the plate. The whole procedure, from aspiration of media to addition of 80% methanol solution, took about 15 sec. The plate was transferred on dry ice to –80 °C freezer and incubated for 15 min. Cells were collected in a centrifuge tube with a cell scraper, sonicated to break up the cells to release metabolites, and then centrifuged at 13,500 × *g* for 10 min, keeping the whole process at a low temperature. Supernatant was collected and stored on dry ice. The cell pellet was resuspended in 0.5 ml of 80% Methanol: 20% Water (spiked with 20 μM 5-Fluorouracil) at 4 °C. The pellet was re-extracted one more time and all the supernatants were combined and stored at –80 °C overnight. The supernatant was evaporated down to 1 ml using a refrigerated rotary evaporator, deproteinized by centrifugation through a 10 kD cut-off filter membrane, and further evaporated (but not completely dried) and then placed in –80 °C freezer. Samples were diluted with 200 μL of mobile phase A (25% ACN 5 mM Ammonium Acetate) and then centrifuged at 20,000 × *g* for 10 min to remove insoluble particles for LC-MS detection of Metabolites.

Metabolites were analyzed by LC-MS on an Ultimate 3000 UHPLC liquid chromatography system (ThermoScientific) equipped with a C18 column (Phenomenex, 00B-4252-B0) and a TSQ Quantiva mass spectrometer (Thermo Scientific). 25% ACN and 5mM Ammonium Acetate buffer (mobile phase A) and 50% IPA, 45% ACN, and 5mM ammonium acetate (mobile phase B) were used at a flow rate of 0.300mL/min. Metabolites were detected by a gradient method, starting with 2% of eluent B, and after 2 min rising to 99% of B, decreasing to 2% B after 6 min. The MS settings were as follows: the nebulizing gas was nitrogen at a pressure of 0.28 MPa; the ion spray voltage was 400 V; the drying gas temperature was 350; the drying gas flow rate was 12 L/min; and the detection mode was multiple reaction monitoring (MRM).

### Cholesterol measurement

The total cellular cholesterol level was quantified using the Amplex Red cholesterol assay kit (Invitrogen). To quantify the intracellular cholesterol, tumor cells were fixed with 0.1% glutaraldehyde and then treated with 2 U/ml cholesterol oxidase for 15 min to oxidize the plasma membrane cholesterol. The intracellular cholesterol was then extracted with methanol/chloroform (vol/vol, 1: 2), and quantified using the Amplex Red cholesterol assay kit. The value of the plasma membrane cholesterol was obtained by subtracting the intracellular cholesterol from the total cellular cholesterol.

### Stable isotope tracing of plasma membrane cholesterol

H1299 cells were plated in 10 cm dishes and cultured in DMEM containing 1% lipoprotein deficient serum (LPDS) and 1% penicillin/streptomycin. Cells were grown to 10-20% confluence, then the medium was replaced with fresh medium containing 5 mM ^13^C-acetate for 72 h. After labeling, the medium was removed, and the cells were washed with PBS. Following trypsinization, cells were collected, centrifuged at 100 × *g* for 10 min at 4 °C. The cell pellet was biotinylated by 1 mg/ml sulfo-NHS-Sbiotin, and then the cells were lysed with a ball-bearing homogenizer. The plasma membrane was isolated from the supernatant of the homogenate using streptavidin magnetic beads.

Cell membrane samples enriched using magnetic beads were homogenized in 1 × PBS. Add 2 mL of hexane:IPA (3:2) and 20 μL acetic acid, then centrifuge at 2000 × *g* for 5 min at 4 °C. Transfer the supernatant to a new tube and dry it using a nitrogen blower. Add 200 μL of 8 M KOH and hydrolyze at 55 °C for 1 h with shaking. Cool to room temperature and adjust pH to 3.0 with HCl. Add 1 mL of hexane and 2 mL of IPA:hexane: acetic acid (40:10:1), followed by 1 mL of water. Vortex and centrifuge at 600 × *g* for 3 min. Extract the upper organic phase and repeat the extraction twice with 1 mL hexane each time.

Dry the pooled supernatant using a nitrogen blower. Add 40 μL of pyridine and 40 μL of BSTFA, then heat at 90 °C for 1 h in a metal bath. Centrifuge at 12,000 × *g* for 10 min at 4 °C, and analyze the supernatant using GC-MS (QP-2010 Ultra, Shimadzu, Japan) on an HP-5ms column (30 m × 0.25 mm × 0.25 μm). The inlet temperature was set to 250 °C. The temperature program was as follows: start at 110 °C, heat to 250 °C at 30 °C/min for 1 min, then to 280 °C at 10 °C/min, and finally to 300 °C at 3 °C/min.

Characteristic cholesterol fragment peaks were detected at 329, 368, and 465. The incorporation of ^13^C-acetate into cholesterol was expressed as M + 2/M + 4/M + 6/M + 8, corrected for natural isotope abundances.

### Modulation of the plasma membrane cholesterol level

To deplete cholesterol from the plasma membrane, tumor cells were treated with 0.1 mM MβCD for 5 min at 37 °C, and then washed three times with PBS. To add cholesterol to the plasma membrane, tumor cells were incubated with culture medium containing 10 μg/ml MβCD-coated cholesterol at 37 °C for 15 min. The cells were then washed three times with PBS.

### Generation of CAR-T cells

Human PBMCs were purchased from Milestone Biotechnologies (Shanghai, China). The generation of CAR-T cells from PBMCs was performed as described previously^42^. PBMCs were resuspended at 1 × 10^6^ cells/mL in complete OpTmizer T Cell Expansion SFM medium, supplemented with 5% Human AB serum, 10 mM neutralized NAC (Sigma-Aldrich #A9165), 100 U/mL penicillin/streptomycin, 2 mM L-glutamine, and 10 ng/mL IL-7/IL-15. PBMCs were stimulated with the Human T-Activator CD3/CD28 Dynabeads (Life Technologies #11132D) for 24 h at a 1:1 bead:cell ratio. Then PBMCs were transduced with lentivirus for 24 h. At day 4 after T cells stimulation, the Dynabeads were removed and CAR^+^ T cells were sorted with BD FACSAria III or BD FACSAria SORP for subsequent assays. After sorting and culturing, the percentage of CD3^+^ T cells was up to 95%. CAR-T cells were cultured in normal complete medium in all experiments. CAR surface level and CD4/CD8 ratio were measured around 14 days after initial stimulation of PBMCs by CD3/CD28 Dynabeads.

### In vitro killing assay

A549 or H1299 cells were seeded in 96-well plates at a density of 1 × 10^4^ cells per well and cultured for 24 h. CD19 CAR-T, EGFR CAR-T, or NK-92MI cells were then added to each well at the desired effector-to-target (E:T) ratios and cocultured with the tumor cells for 24 h (for CAR-T cells) or 4 h (for NK-92MI cells). After incubation, the supernatant was removed, and the tumor cells were washed twice with PBS. CellCounting-Lite (Vazyme) reagent was added to each well, and tumor cell viability was assessed using the Ensight high-throughput screening system.

### Subcutaneous xenograft model

In brief, 1 × 10^6^ A549 cells stably expressing the gene of interest were subcutaneously injected into the left dorsal region of randomized 6-week-old female B-NDG mice (six mice per group). 5 days after inoculation, mice were treated with the corresponding reagents. For NK-92MI cell treatment, NK-92MI cells were prepared in PBS solution, irradiated on ice with 10 Gy of X-rays and injected intravenously into these mice (1 × 10^7^ cells per mice). Following NK-92MI cell infusion, recombinant human IL-2 was administered intraperitoneally at 75,000 units per mouse every other day to support NK cell persistence and activity. For CD19 CAR-T cell treatment, CD19 CAR-T cells were prepared in PBS solution and injected intravenously into these mice (1 × 10^7^ cells per mice).

1 × 10^6^ LLC cells stably expressing the gene of interest were subcutaneously injected into the left dorsal region of randomized 6-week-old female C57BL/6 mice (six mice per group). 5 days after inoculation, mice were treated with anti-PD-1 antibody. 200 μg control IgG2a or anti-PD-1 antibody per mouse was administered intraperitoneally every third day.

Tumor volumes were measured using length (a) and width (b) and calculated using the equation: V = ab^2^/2. Data are represented as means ± SD of tumor volume from six mice.

### Orthotopic model of lung cancer

Luciferase-expressing LLC cells were resuspended in DMEM and mixed with an equal volume of growth factor-reduced Matrigel (BD Biosciences). Randomized C57BL/6 mice (6-week-old, female) were anaesthetized, and placed in the right lateral decubitus position. Then, a total volume of 50 μl containing 1 × 10^6^ cells were injected into the left lung of the mice with a 30-gauge needle. 14 days after inoculation, bioluminescence imaging was performed using a Xenogen IVIS system (version 4.0). For PD-1 blockade, 200 μg control IgG2a or anti-PD-1 antibody per mouse was administered intraperitoneally every third day.

### Statistics and reproducibility

No statistical methods were used to pre-determine sample sizes but our sample sizes are similar to those reported in previous publications^4^. All statistics were performed using GraphPad Prism software (version 8.0). Specific statistical tests for each experiment are described in Figure legends. A *p*-value of <0.05 was considered statistically significant.

### Reporting summary

Further information on research design is available in the Nature Portfolio Reporting Summary linked to this article.

## Supplementary information

Supplementary Information

Reporting Summary

Transparent Peer Review file

## Source data

Source data

## Data Availability

The source data underlying Figs. 1–6 and Supplementary Figs. 1–8 are provided as a Source Data file. The data presented in Fig. 6a was obtained from the Gene Expression Omnibus (GEO, https://www.ncbi.nlm.nih.gov/geo/query/acc.cgi) under accession number GSE91061. The data presented in Fig. 6b was derived from GEO under accession number GSE205506. The data underlying Fig. 6g was generated via the Kaplan‑Meier Plotter Immunotherapy module (https://www.kmplot.com/analysis/index.php?p=service&cancer=immunotherapy), which allowed full reproducibility by selecting HMGCS1 under Gene symbol field and All anti‑PD1 under Anti‑PD‑1 treatment field. The metabolite content data generated in this study are also provided in the Source Data file. Source data are provided with this paper.
