# Supplementary material for: HMGCS1 drives cholesterol-dependent membrane repair and shields tumor cells from lymphocyte attack
Source: Nat Commun. 2026 Jun 5;17:7204. doi: 10.1038/s41467-026-74022-y (PMC13396504; doi:10.1038/s41467-026-74022-y)
Supplement: Supplementary file 1 — Supplementary Information [file 41467_2026_74022_MOESM1_ESM.pdf]

## Supplementary Information

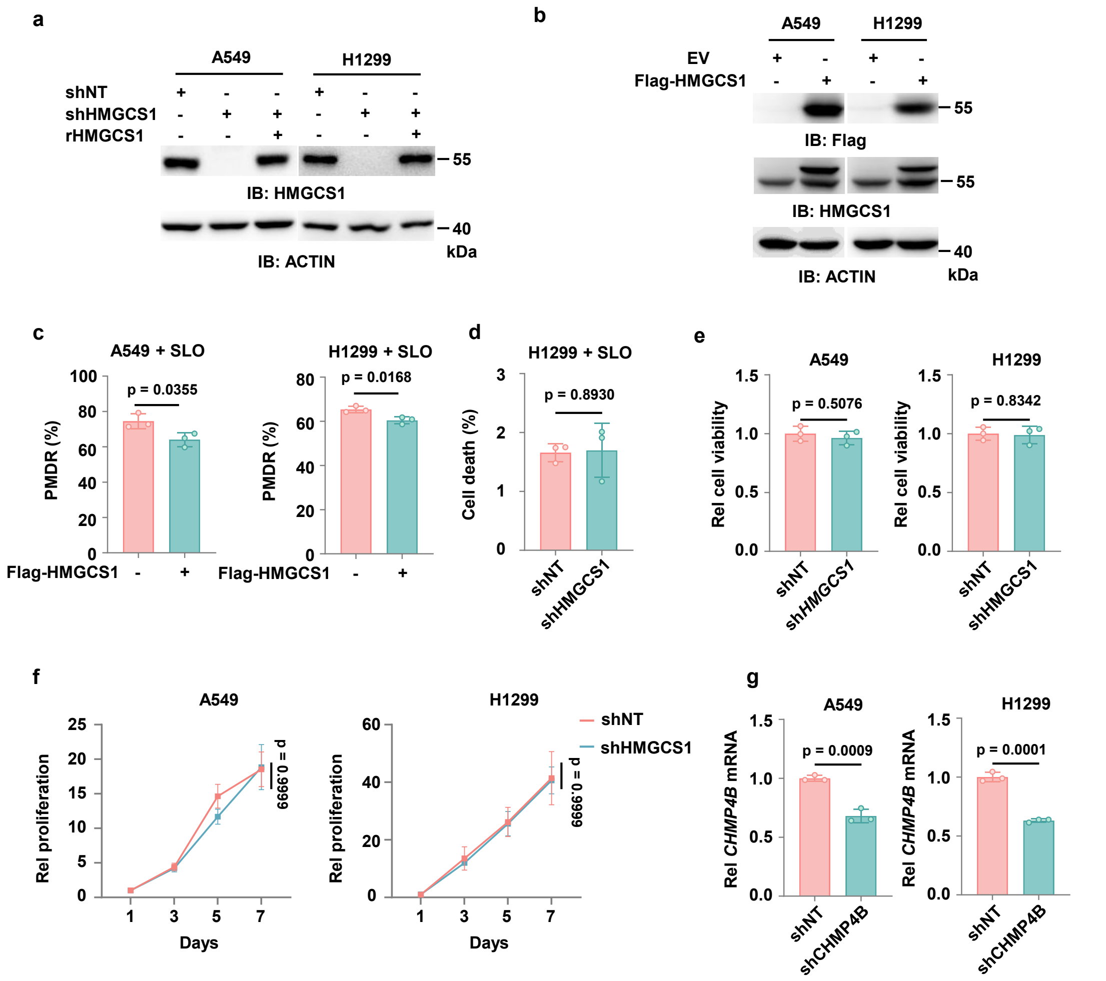


**Supplementary Fig. 1: Functional screen identifies HMGCS1 necessary for PM repair. Related to Fig. 1.**

a. HMGCS1-depleted A549 or H1299 cells were rescued with rHMGCS1 WT. Cells were harvested and subjected to immunoblotting analyses.

b,c. A549 or H1299 cells stably expressing EV or Flag-HMGCS1 were harvested and subjected to immunoblotting analyses (b). Cells were treated with or without SLO. PI and Hoechst staining were performed, and PMDR was calculated (c).

d. H1299 cells stably expressing shNT or shHMGCS1 were treated with SLO for 5 minutes at 37°C. Cells were harvested and stained with FITC-annexin V, followed by flow cytometry analysis.

e,f. HMGCS1 was depleted in A549 or H1299 cells. Cell viability assay with trypan blue

staining (e) and cell proliferation assay (f) were performed in these cells. Rel, relative.

g. A549 or H1299 cells were depleted of CHMP4B. CHMP4B expression was examined.

a,b. Immunoblots are representative of three biologically independent experiments. c-g. Data represent the mean ± s.d. of three biologically independent experiments. c-e,g. Two-tailed Student's t-test. f. Two-way ANOVA with Sidak's multiple comparisons test. Source data are provided as a Source Data file.

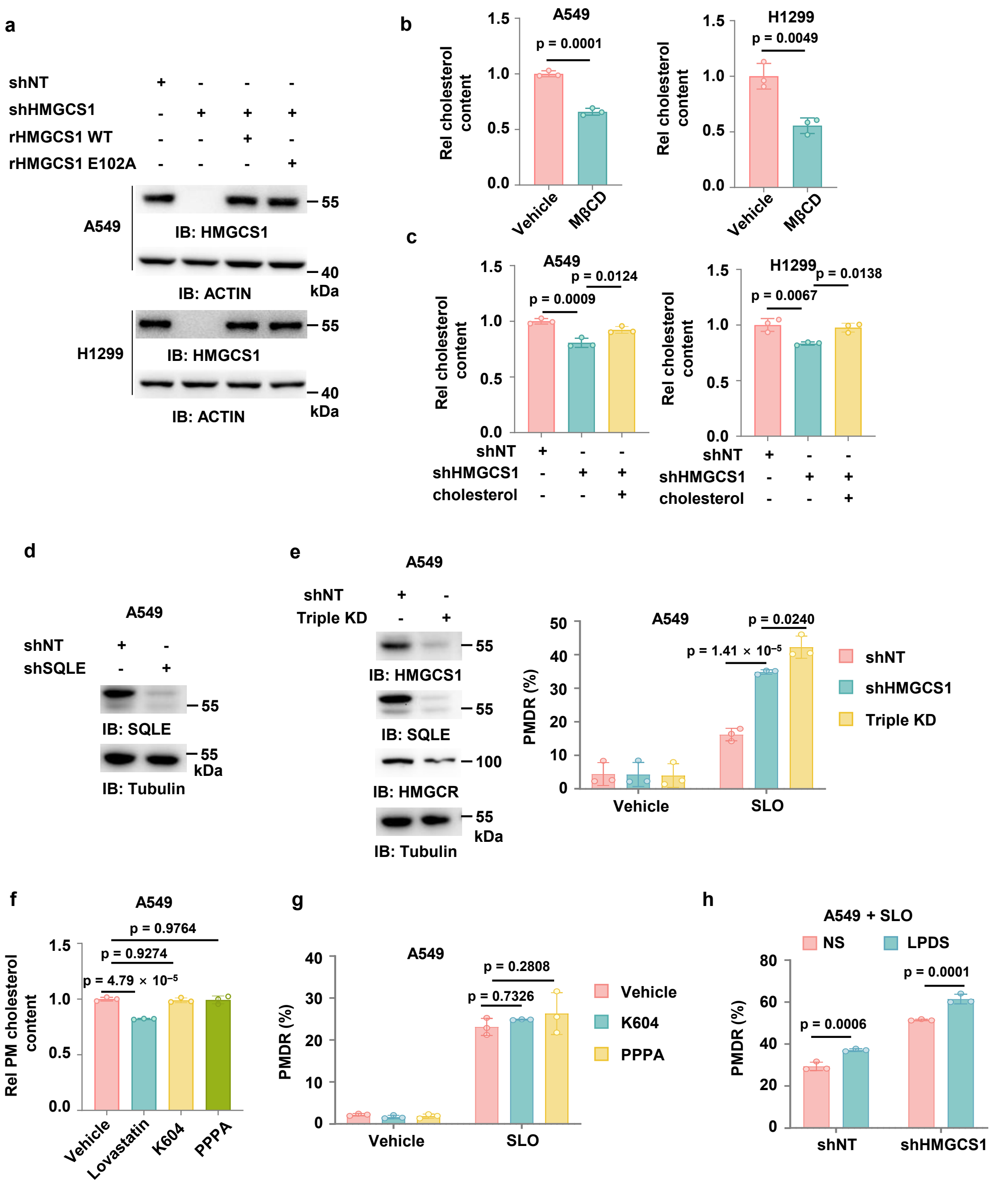


**Supplementary Fig. 2: HMGCS1-dependent *de novo* cholesterol synthesis supports PM repair by producing cholesterol. Related to Fig. 2.**

a. HMGCS1-depleted A549 or H1299 cells were rescued with rHMGCS1 WT or E102A. Cells were harvested and subjected to immunoblotting analyses.

b. Cholesterol was depleted using MβCD in A549 or H1299 cells. Cells were harvested and cholesterol level was examined.

c. HMGCS1-depleted A549 or H1299 cells were rescued with MβCD-coated cholesterol. Cells

were harvested and cholesterol level was examined.

d. A549 cells were depleted of SQLE. Cells were harvested and subjected to immunoblotting analyses.

e. A549 cells were depleted of HMGCS1, SQLE and HMGCR. Cells were harvested and subjected to immunoblotting analyses (left). Cells were treated with or without SLO, and PMDR was calculated (right).

f. A549 cells were treated with 10 μM Lovastatin, K604 or PPPA for 12 h. Cells were harvested and PM cholesterol level was examined.

g. A549 cells were treated with 10 μM K604 or PPPA for 12 h. Cells were treated with or without SLO.

h. A549 cells were cultured in medium containing either normal serum (NS) or lipoprotein-deficient serum (LPDS). Cells were treated with or without SLO, and PMDR was calculated.

a,d,e. Immunoblots are representative of three biologically independent experiments. b,c,e-h. Data represent the mean ± s.d. of three biologically independent experiments. b. Two-tailed Student's t-test. c,f. One-way ANOVA with Tukey's multiple comparisons test. e,g. Two-way ANOVA with Tukey's multiple comparisons test. h. Two-way ANOVA with Sidak's multiple comparisons test. Source data are provided as a Source Data file.

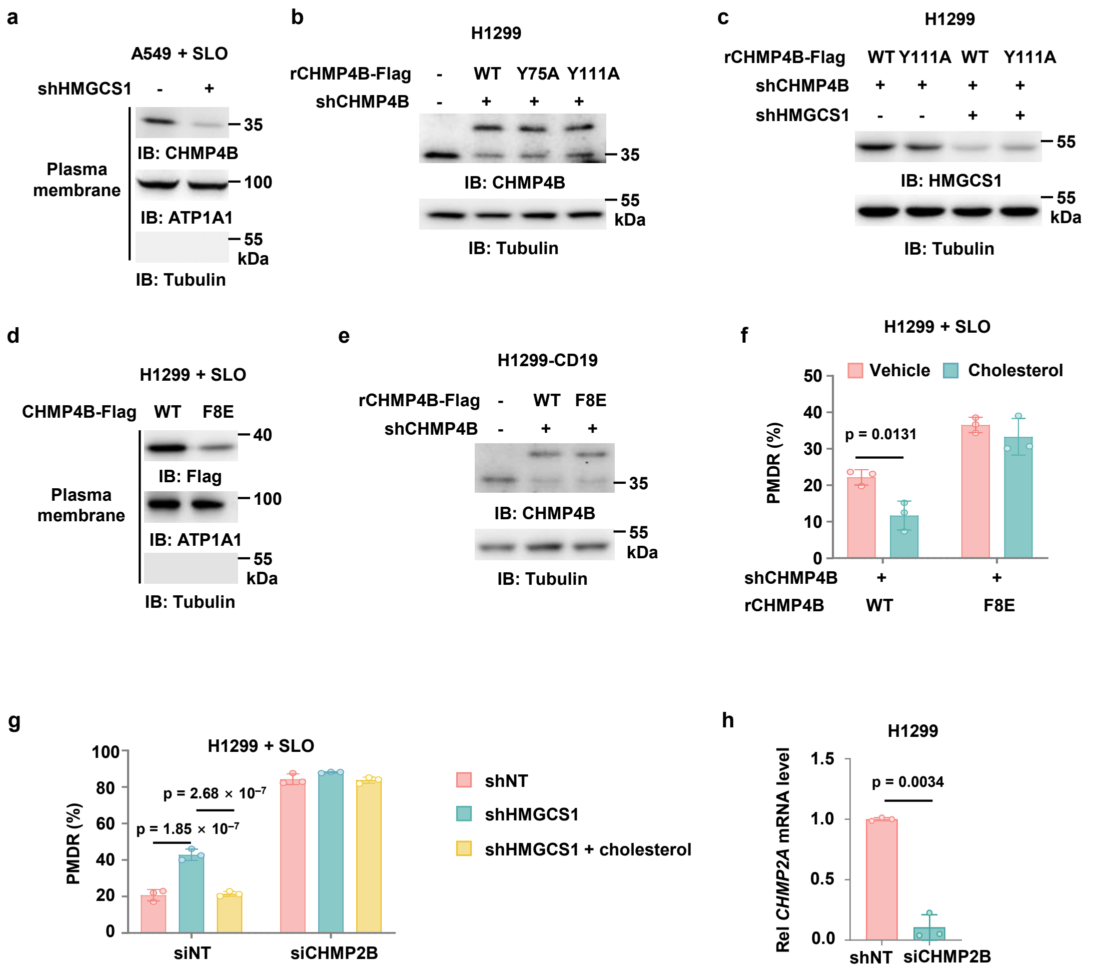


**Supplementary Fig. 3: Cholesterol supports PM repair by promoting membrane localization of CHMP4B and providing building blocks. Related to Fig. 3.**

a. HMGCS1-depleted A549 cells were treated with SLO. Cells were harvested and cell plasma membrane was isolated to subject to immunoblotting analyses. ATP1A1 was used as a loading control for membrane proteins.

b. CHMP4B-depleted H1299 cells were rescued with rCHMP4B WT, Y75A or Y111A. Cells were harvested and subjected to immunoblotting analyses.

c. HMGCS1 was depleted in CHMP4B-depleted H1299 cells rescued with rCHMP4B WT or Y111A. Cells were harvested and subjected to immunoblotting analyses.

d. CHMP4B-Flag WT or F8E-transfected H1299 cells were treated with SLO. Cells were harvested and cell plasma membrane was isolated to subject to immunoblotting analyses.

e,f. CHMP4B-depleted H1299-CD19 cells were rescued with rCHMP4B WT or F8E. Cells

were harvested and subjected to immunoblotting analyses (e). Cells were treated with SLO and PMDR was calculated (f).

g,h. HMGCS1-depleted H1299 cells were transfected with siNT or siCHMP2B. Cells were rescued with or without MβCD-coated cholesterol before treated with SLO. PMDR was calculated (g). The efficacy of siCHMP2B was examined (h).

a-e. Immunoblots are representative of three biologically independent experiments. f-h. Data represent the mean ± s.d. of three biologically independent experiments. f. Two-way ANOVA with Sidak's multiple comparisons test. g. Two-way ANOVA with Tukey's multiple comparisons test. h. Two-tailed Student's t-test. Source data are provided as a Source Data file.

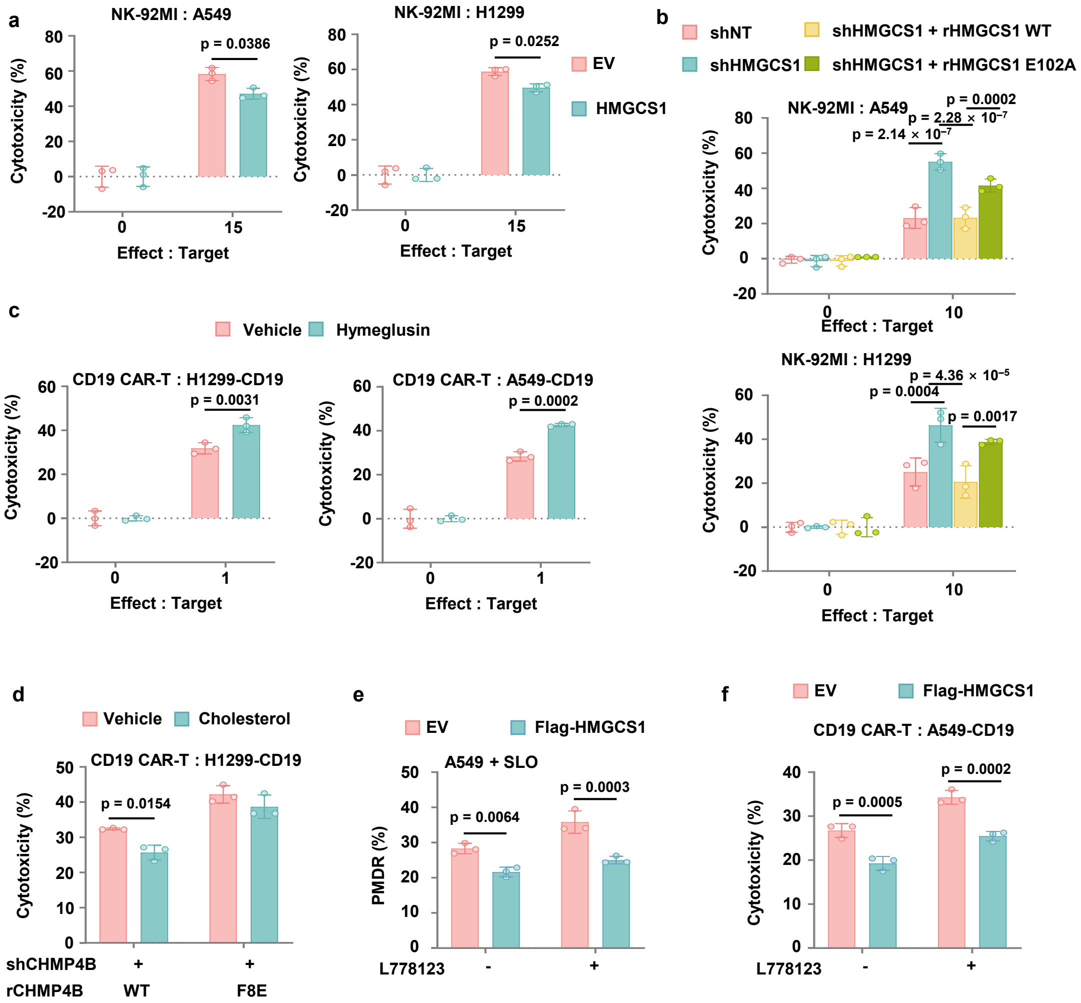


**Supplementary Fig. 4: HMGCS1-promoted PM repair prevents tumor cells from lymphocyte killing. Related to Fig. 4.**

a. Flag-HMGCS1 was overexpressed in A549 or H1299 cells. Cells were cocultured with NK-92MI cells for 4 h.

b. HMGCS1-depleted A549 cells were rescued with rHMGCS1 WT or E102A. Cells were cocultured with NK-92MI cells for 4 h.

c. HMGCS1-depleted H1299-CD19 or A549-CD19 cells were co-cultured with CD19 CAR-T cells for 24 h in the presence or absence of 10 μM hymeglusin.

d. CHMP4B-depleted H1299-CD19 cells were rescued with rCHMP4B WT or F8E. Cells were pre-treated with MβCD-coated cholesterol before cocultured with CD19 CAR-T for 24 h.

e. HMGCS1-overexpressed A549 cells were pre-treated with or without 10 μM L778123 for 12

h before treated with SLO.

f. HMGCS1-overexpressed A549-CD19 cells were pre-treated with or without 10 μM L778123 for 12 h before cocultured with CD19 CAR-T for 24 h.

a-f. Data represent the mean ± s.d. of three biologically independent experiments. a,c-f. Two-way ANOVA with Sidak's multiple comparisons test. b. Two-way ANOVA with Tukey's multiple comparisons test. Source data are provided as a Source Data file.

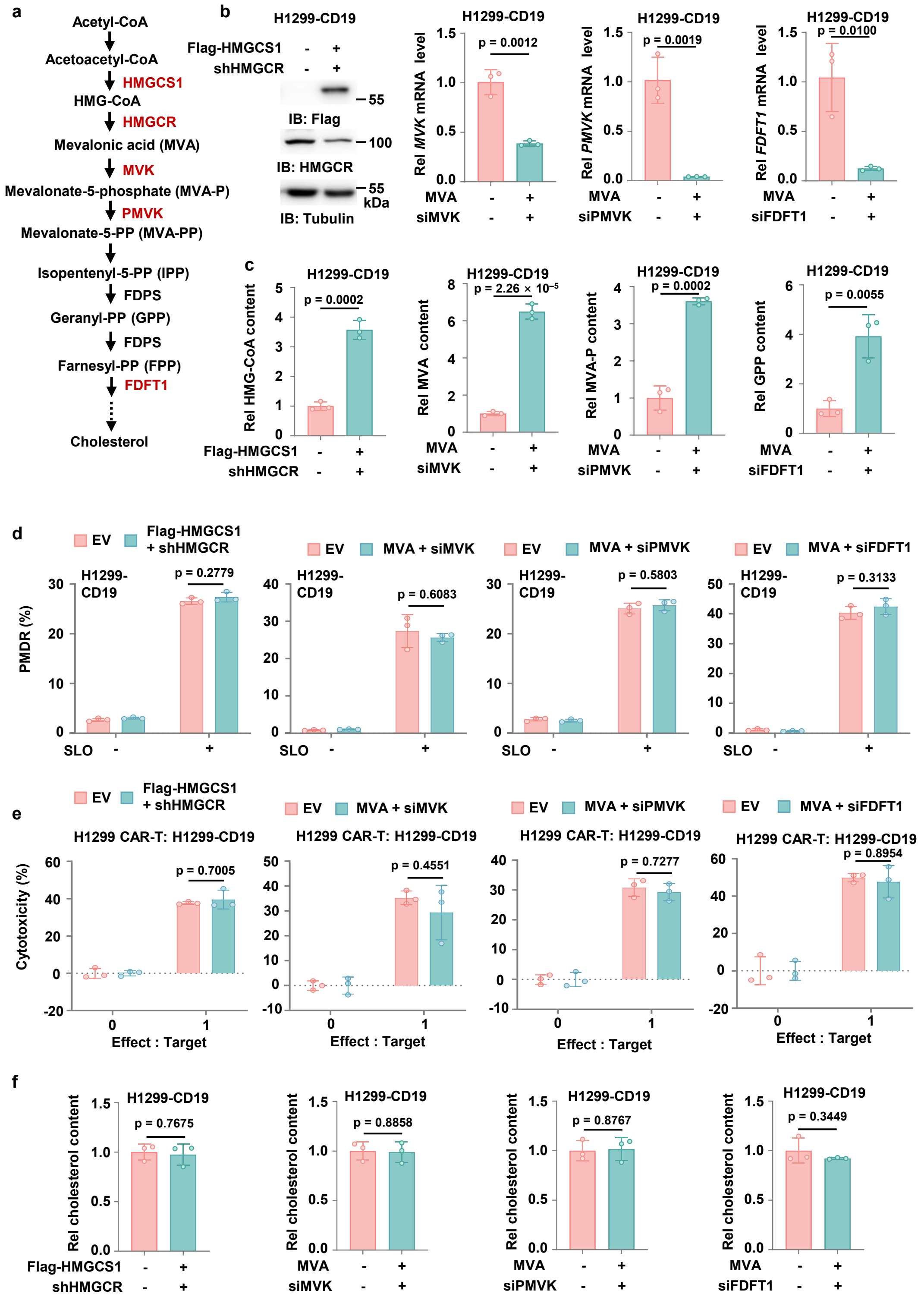


**Supplementary Fig. 5: HMGCS1 confers tumor cells the resistance to immune killing through cholesterol. Related to Fig. 4.**

a. Schematic diagram of the mevalonic acid and cholesterol synthesis pathway.

b,c. H1299-CD19 cells were manipulated to elevate specific metabolite levels through genetic

and pharmacological interventions: HMGCS1 overexpression coupled with HMGCR knockdown to increase HMG-CoA; exogenous mevalonate (MVA) supplementation (200 μM) combined with MVK knockdown to raise MVA levels; MVA supplementation with PMVK knockdown to elevate MVA-P; and MVA supplementation with FDFT1 knockdown to increase GPP. The knockdown efficiency of HMGCR, MVK, PMVK, and FDFT1 was confirmed in (b), and the corresponding levels of HMG-CoA, MVA, MVA-P, and GPP were measured in (c).

d. Cells in (b) were treated with or without SLO, and PMDR was measured.

e. Cells in (b) were cocultured with or without CD19 CAR-T for 24 h.

f. Cholesterol levels were measured in cells in (b).

b. Immunoblots are representative of three biologically independent experiments. b-f. Data represent the mean ± s.d. of three biologically independent experiments. b,c,f. Two-tailed Student's t-test. d,e. Two-way ANOVA with Sidak's multiple comparisons test. Source data are provided as a Source Data file.

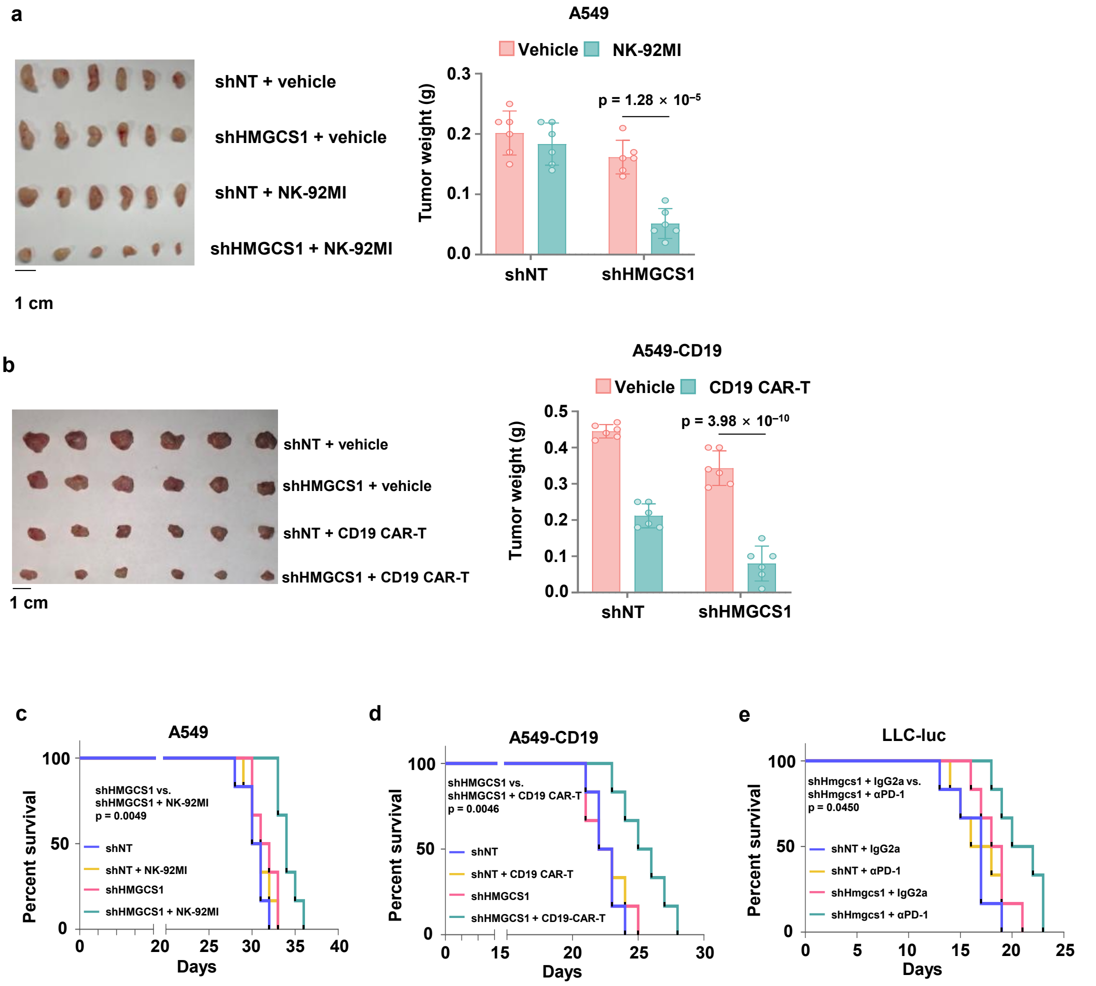


**Supplementary Fig. 6: HMGCS1 depletion enhances the efficacy of immune therapy. Related to Fig. 4.**

a. HMGCS1-depleted A549 cells were subcutaneously injected into female B-NDG mice. 5 days after inoculation, mice were treated with NK-92MI cells. 19 days after inoculation, tumors were dissected and tumor weight was measured.

b. HMGCS1-depleted A549-CD19 cells were subcutaneously injected into female B-NDG mice. 5 days after inoculation, mice were treated with CD19 CAR-T cells. 17 days after inoculation, tumors were dissected and tumor weight was measured.

c. HMGCS1-depleted A549 cells were subcutaneously injected into female B-NDG mice (six mice per group). 5 days after inoculation, mice were treated with NK-92MI cells. Kaplan-Meier survival analysis with the log-rank (Mantel-Cox) test was performed.

d. HMGCS1-depleted A549-CD19 cells were subcutaneously injected into female B-NDG

mice (six mice per group). 5 days after inoculation, mice were treated with CD19 CAR-T cells. Kaplan-Meier survival analysis with the log-rank (Mantel-Cox) test was performed.

e. Hmgcs1-depleted LLC cells stably expressing luciferase were injected into lung of female C57BL/6 mice (six mice per group). 5 days after inoculation, mice were treated with control IgG2a or anti-PD-1 antibody by intraperitoneal injection. Kaplan-Meier survival analysis with the log-rank (Mantel-Cox) test was performed.

a,b. Data represent the mean ± s.d. of six mice. Two-way ANOVA with Sidak's multiple comparisons test. c,d,e. n = 6 / group, log-rank (Mantel-Cox) test. Source data are provided as a Source Data file.

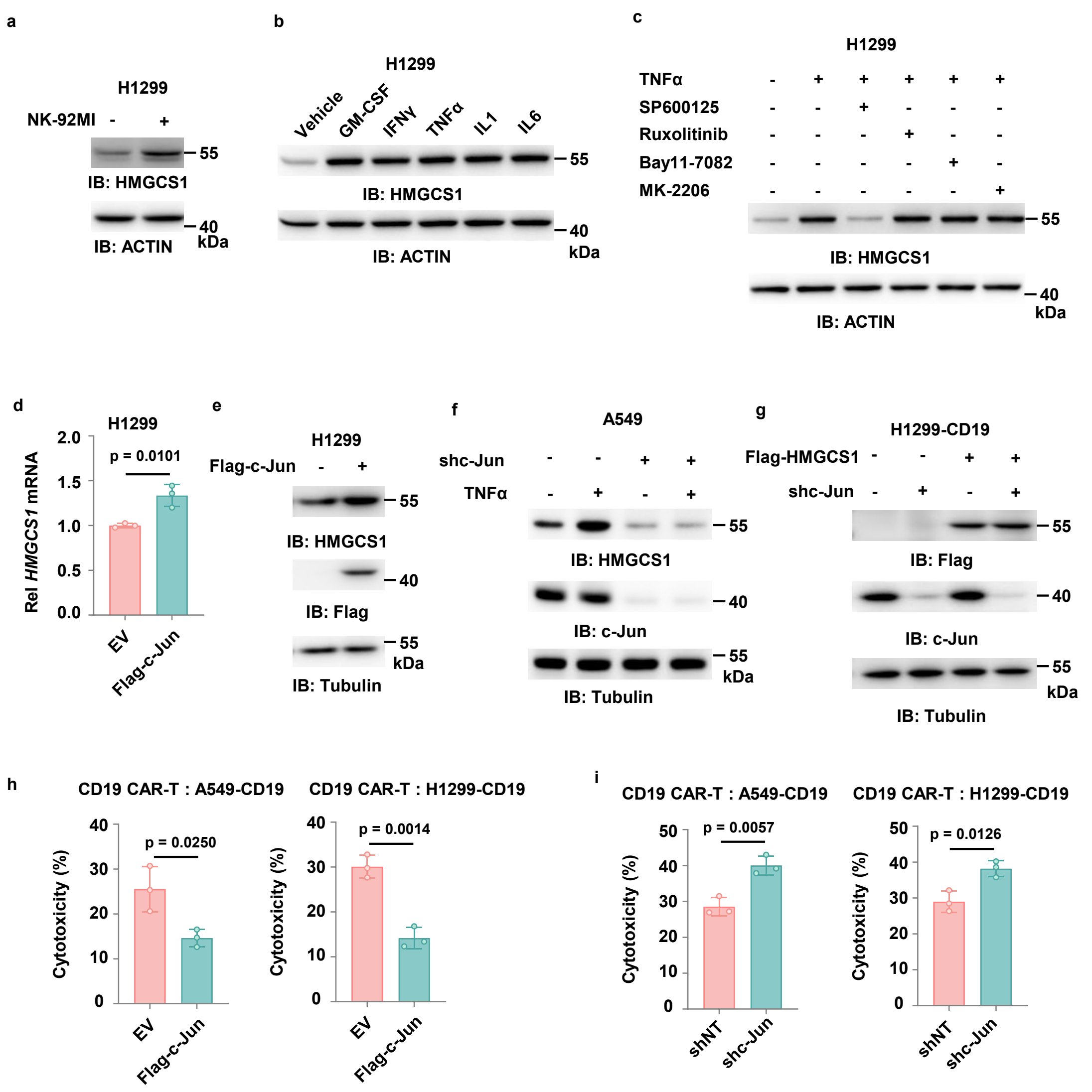


**Supplementary Fig. 7: HMGCS1 expression is upregulated by c-Jun. Related to Fig. 5.**

a. H1299 cells were cocultured with NK-92MI cells for 12 h. H1299 cells were harvested and subjected to immunoblotting analyses.

b. H1299 cells were treated with 20 ng/ml GM-CSF, IFNγ、TNFα、IL1 or IL6 for 12 h. Cells were harvested and subjected to immunoblotting analyses.

c. H1299 cells were pre-treated with 10 μM SP600125, Ruxolitinib, Bay11-7082, or MK-2206 for 12 h, followed by treatment with 20 ng/ml TNFα for an additional 12 h.

d,e. H1299 cells expressing EV or Flag-c-Jun were harvested and HMGCS1 expression in these cells was examined.

f. c-Jun-depleted A549 cells were treated with or without TNFα. Cells were harvested and subjected to immunoblotting analyses.

g. c-Jun-depleted H1299-CD19 cells were overexpressed with EV or Flag-HMGCS1. Cells were harvested and subjected to immunoblotting analyses.

h. Flag-c-Jun-overexpressed A549-CD19 or H1299-CD19 cells were cocultured with CD19 CAR-T cells for 24 h.

i. c-Jun-depleted A549-CD19 or H1299-CD19 cells were cocultured with CD19 CAR-T cells for 24 h.

a-c,e-g. Immunoblots are representative of three biologically independent experiments. d,h,i. Data represent the mean ± s.d. of three biologically independent experiments. Two-tailed Student's t-test. Source data are provided as a Source Data file.

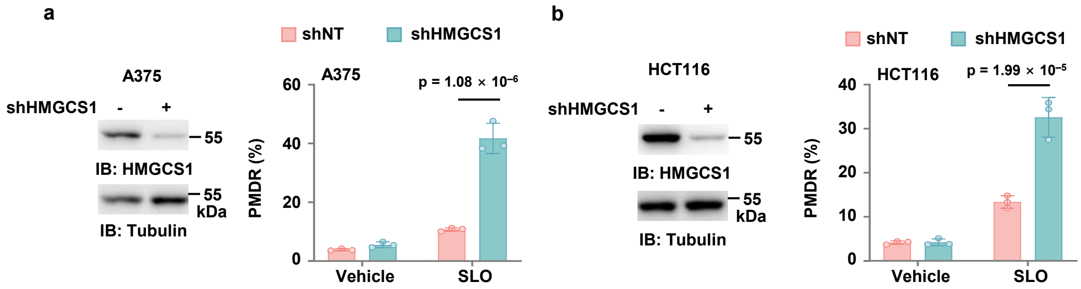


**Supplementary Fig. 8: HMGCS1 depletion sensitizes tumor cell to SLO-induced PM damage. Related to Fig. 6.**

a. HMGCS1 was depleted in A375 cells. Cells were harvested and subjected to immunoblotting analyses (left). Cells were treated with or without SLO and PMDR was examined (right).

b. HMGCS1 was depleted in HCT116 cells. Cells were harvested and subjected to immunoblotting analyses (left). Cells were treated with or without SLO and PMDR was examined (right).

a,b. Immunoblots are representative of three biologically independent experiments. Data represent the mean ± s.d. of three biologically independent experiments. Two-way ANOVA with Sidak's multiple comparisons test. Source data are provided as a Source Data file.

| | Protein Name | Amino Acid Position | Amino Acid Sequence |
|---|---|---|---|
| ESCRT-0 | HGS | 127 - 136 | VvqdtYqimK |
| | | 211 - 222 | VcepcYeqlnrK |
| | | 280 - 291 | LrqkstYtsypK |
| | | 326 - 332 | LarYlnR |
| | | 330 - 338 | LnrnYwekK |
| | | 465 - 473 | LyYeglqdK |
| | STAM1 | 368 - 373 | LslYtK |
| | STAM2 | 355 - 363 | LealelYnK |
| | | 365 - 376 | VneapvYsvysK |
| ESCRT-I | TS101 | 12-23 | VskykYrdltvR |
| | | 26 - 36 | VnvitlYkdlK |
| | | 111 - 118 | LpYlhewK |
| | VPS28 | 24 - 33 | VklYknareR |
| | | 52 - 58 | LekaYiK |
| | | 61 - 72 | VspseYtaacsR |
| | | 73 - 82 | LlvqYkaafR |
| | | 208 - 217 | LesaYnafnR |
| | VP37A | 79 - 87 | VisvYppiR |
| | VP37B | 82 - 93 | VlfeaYqikktK |
| | VP37C | 67 - 76 | LsdrYqelrK |
| | VP37D | 58 - 66 | LasnYalaK |
| | | 82 - 90 | LaikYqelR |
| | UBAP1 | 48 - 60 | VvrevqYdfsleK |
| | | 400 - 411 | VnmgysYecvlR |
| | UBP2L | 401 - 407 | LvqYdlK |
| | | 853 - 864 | LasnpYsgdltK |
| ESCRT-II | VPS22 | 16 - 24 | LaeakYkeR |
| | VPS25 | 110 - 117 | LiYqwvsR |
| | VPS36 | 24 - 32 | VriYdgeeK |
| | | 266 - 275 | LtevYclvnR |
| ESCRT-III | CHMP6 | 30 - 36 | LrqYqkR |
| | | 134 - 138 | VeYqR |
| | CHMP4A | 48 - 60 | LqtakkYgtknkR |
| | CHMP4C | 36 - 46 | LgkkqeYlenR |
| | CHMP3 | 74 - 81 | VsklYasK |
| | CHMP2A | 66 - 75 | LvrtrrYvrK |
| | CHMP2B | No hit. | |
| | CHMP1A | 42 - 54 | VecarvYaenaiR |
| | CHMP1B | No hit. | |
| | IST1 | 124 - 134 | LcakYskeygK |
| | | 161 - 171 | LverYlieiaK |
| | CHMP7 | No hit. | |

**Supplementary Fig. 9: Cholesterol-recognition motif in ESCRT.**

A systematic in silico analysis was performed to examine core ESCRT proteins for the presence of established cholesterol-recognition motifs, (L/V)-$X_{1-5}$-Y-$X_{1-5}$-(K/R).

**Supplementary Table 1. The list of genes encoding 111 key metabolic enzymes. Related to Figure 1.**

| | | | |
|---|---|---|---|
| *ACADL* | *DCK* | *LIPE* | *SAT2* |
| *ACO1* | *DDC* | *LPCAT2* | *SCD* |
| *ACO2* | *DLD* | *LTA4H* | *SCD5* |
| *ACSL1* | *DLST* | *LTC4S* | *SOAT1* |
| *ADH1A* | *DPYD* | *MYLK* | *SOAT2* |
| *ADH7* | *DTYMK* | *MYLK2* | *SPTLC1* |
| *ADK* | *F2* | *MYLK3* | *SPTLC2* |
| *ALAD* | *FBP1* | *NAT1* | *SQLE* |
| *ALAS1* | *FBP2* | *NAT2* | *STS* |
| *ALAS2* | *G6PC* | *OGDH* | *TAT* |
| *ALDH1A1* | *G6PC2* | *OGDHL* | *TK1* |
| *ALDH1A2* | *G6PD* | *PAH* | *TPH1* |
| *ALDH1A3* | *GAD1* | *PCK1* | *TPH2* |
| *ALDH1B1* | *GAD2* | *PCK2* | *TYMP* |
| *ALDH2* | *GGT1* | *PIK3C3* | *TYR* |
| *ALDH3A1* | *GNE* | *PKLR* | *UCK1* |
| *ALDH3A2* | *GPAM* | *PKM2* | *UCK2* |
| *ALDH7A1* | *GPD2* | *PLAT* | *UCKL1* |
| *ALDH9A1* | *HDC* | *PLAU* | *UGDH* |
| *ALOX5* | *HK1* | *PTGS1* | *UGT2B4* |
| *APRT* | *HK2* | *PTGS2* | *XDH* |
| *ASS1* | *HK3* | *PYGB* | |
| *BACE1* | *HMGCR* | *PYGL* | |
| *CHAT* | *HMGCS1* | *PYGM* | |
| *COX4I1* | *HMGCS2* | *RDH5* | |
| *COX5B* | *HSD17B6* | *REN* | |
| *COX6A1* | *HSD3B1* | *RRM1* | |
| *COX6A2* | *HSD3B2* | *RRM2* | |
| *COX6B1* | *IMPDH1* | *RRM2B* | |
| *COX6C* | *IMPDH2* | *SAT1* | |

**Supplementary Table 2. Results of the siRNA screen for identifying the metabolic enzyme that supports PM repair. Related to Figure 1.**

The fold change represents the ratio of the PMDR scores in the siRNA-treated group to that in the non-targeting siRNA control, with each data point calculated as the mean of three biological replicates. Statistical significance was determined using a two-tailed Student's t-test.

| **gene name** | **fold change** | **p value** |
|---|---|---|
| NAT2 | 2.841805665 | 1.65653E-17 |
| SCD | 2.598546364 | 1.90993E-10 |
| HMGCS1 | 2.523037589 | 4.09272E-14 |
| ALDH1B1 | 2.480924618 | 1.44325E-09 |
| HMGCS1 | 2.473204267 | 6.51046E-14 |
| LTA4H | 2.471310355 | 5.04796E-13 |
| SPTLC2 | 2.417807525 | 4.19835E-13 |
| DLD | 2.404220138 | 4.51224E-09 |
| RDH5 | 2.374295663 | 8.69507E-09 |
| CHAT | 2.341696168 | 1.96803E-11 |
| SOAT1 | 2.329977624 | 2.0061E-11 |
| ALDH7A1 | 2.328370752 | 1.84136E-08 |
| SCD | 2.28476645 | 3.49453E-08 |
| HMGCS1 | 2.279315604 | 3.62603E-11 |
| ALDH3A2 | 2.265956711 | 6.44155E-08 |
| ALDH1B1 | 2.253742671 | 6.42036E-08 |
| HK2 | 2.228093075 | 1.06608E-07 |
| PYGM | 2.201902213 | 2.73504E-10 |
| ALDH1A2 | 2.195036759 | 8.61781E-11 |
| IMPDH2 | 2.18925241 | 2.8124E-07 |
| SCD5 | 2.146387116 | 5.49044E-10 |
| ALAS2 | 2.137746079 | 3.66963E-10 |
| MYLK | 2.131968319 | 6.22305E-07 |
| UCKL1 | 2.115234921 | 6.87329E-07 |
| TYR | 2.103265243 | 1.3395E-06 |
| PTGS2 | 2.102043755 | 9.23431E-07 |
| GNE | 2.097768823 | 1.07182E-06 |
| GGT1 | 2.088608223 | 1.15205E-06 |
| GAD1 | 2.072051269 | 1.48107E-09 |
| ALDH2 | 2.061859461 | 4.28243E-06 |
| DTYMK | 2.060149451 | 2.19944E-06 |
| ACSL1 | 2.05678173 | 8.64175E-09 |
| COX6B1 | 2.048179703 | 3.17164E-06 |
| ACSL1 | 2.040920237 | 8.50937E-09 |
| LIPE | 2.033936446 | 5.84132E-08 |
| ALDH9A1 | 2.033766993 | 2.76869E-06 |
| SAT1 | 2.003633864 | 2.51526E-08 |
| ADH1A | 1.999254313 | 1.34116E-08 |
| PYGM | 1.992270442 | 2.13688E-08 |
| TPH2 | 1.983567235 | 8.63371E-06 |
| UCKL1 | 1.98344501 | 1.03172E-05 |
| PKM | 1.98088002 | 9.1636E-06 |
| ADH7 | 1.961459708 | 9.98211E-06 |
| PCK2 | 1.952176912 | 1.26487E-05 |
| PLAU | 1.942405753 | 1.77359E-05 |
| PKLR | 1.941062134 | 2.44454E-05 |
| SPTLC1 | 1.931665515 | 7.47822E-08 |
| SPTLC1 | 1.924918414 | 2.15508E-07 |
| PTGS2 | 1.921031 | 2.33788E-05 |
| DPYD | 1.919076869 | 2.36076E-05 |

| | | |
|---|---|---|
| PKLR | 1.915534752 | 2.52349E-05 |
| ALOX5 | 1.915330556 | 9.39784E-07 |
| GPAM | 1.904440612 | 3.78799E-07 |
| REN | 1.889640905 | 3.80606E-05 |
| G6PC2 | 1.884755262 | 4.23842E-05 |
| TPH2 | 1.879869612 | 3.90871E-05 |
| TK1 | 1.868510538 | 5.0082E-05 |
| HMGCS1 | 1.850922257 | 6.55959E-05 |
| ACADL | 1.846203008 | 7.08084E-07 |
| BACE1 | 1.810737856 | 0.000206284 |
| TPH1 | 1.792294609 | 0.000196754 |
| TK1 | 1.78997396 | 0.000180194 |
| NAT1 | 1.787491895 | 3.22418E-06 |
| DLST | 1.763226241 | 1.20168E-05 |
| ACSL1 | 1.753638406 | 4.48541E-06 |
| ALDH3A2 | 1.750522494 | 0.000345041 |
| SCD | 1.74233906 | 0.000359668 |
| HSD3B1 | 1.737453419 | 0.000389815 |
| SCD5 | 1.733633936 | 1.16353E-05 |
| COX5B | 1.727071367 | 0.000643404 |
| GPD2 | 1.726295093 | 1.34334E-05 |
| NAT2 | 1.725229751 | 2.85219E-05 |
| GGT1 | 1.721208655 | 0.000932073 |
| ALAD | 1.719666493 | 2.37463E-05 |
| HMGCR | 1.709727377 | 0.000605018 |
| ALDH9A1 | 1.694704104 | 0.000803164 |
| XDH | 1.686996523 | 3.88272E-05 |
| ALDH1B1 | 1.685787862 | 0.001211661 |
| GGT1 | 1.682978563 | 0.001104925 |
| MYLK3 | 1.678459353 | 0.001124396 |
| MYLK | 1.666245293 | 0.001171978 |
| HSD17B6 | 1.6622574 | 4.65917E-05 |
| TYR | 1.654153365 | 0.001894048 |
| ALDH3A2 | 1.648779189 | 0.00162015 |
| DDC | 1.642252999 | 0.000122408 |
| UCK2 | 1.638641434 | 0.002059529 |
| GAD1 | 1.627930377 | 0.000176911 |
| RRM1 | 1.614317889 | 0.000175808 |
| G6PD | 1.610411746 | 0.000179864 |
| TPH2 | 1.603587081 | 0.003659355 |
| SAT2 | 1.599434264 | 0.003287019 |
| RRM2 | 1.571823471 | 0.000543559 |
| PLAU | 1.569265436 | 0.005511822 |
| SQLE | 1.559749774 | 0.00057768 |
| ALDH1A1 | 1.557500795 | 0.00084054 |
| PKM | 1.549845066 | 0.006486671 |
| ALDH2 | 1.549478675 | 0.006770827 |
| NAT2 | 1.547676128 | 0.000701336 |
| DCK | 1.542760913 | 0.009140749 |
| PAH | 1.512958484 | 0.013468818 |
| HDC | 1.507549005 | 0.002200093 |
| ALAD | 1.49701409 | 0.002472719 |
| FBP2 | 1.489996044 | 0.016603349 |
| SPTLC1 | 1.480679196 | 0.002039155 |
| XDH | 1.475707712 | 0.00216324 |
| OGDHL | 1.475470933 | 0.002749278 |
| NAT1 | 1.472038291 | 0.004181667 |
| HMGCR | 1.464102238 | 0.020619575 |

| | | |
|---|---|---|
| SOAT2 | 1.462450405 | 0.004874068 |
| ALDH1A3 | 1.458780884 | 0.004249916 |
| HSD3B1 | 1.458070724 | 0.004967652 |
| GGT1 | 1.457017999 | 0.027620339 |
| UCKL1 | 1.455552322 | 0.022716133 |
| SPTLC2 | 1.453809462 | 0.004381537 |
| PTGS1 | 1.441499064 | 0.00434267 |
| GPAM | 1.440196933 | 0.011688678 |
| ALDH1A1 | 1.433094852 | 0.007223006 |
| GPAM | 1.43214791 | 0.010489754 |
| UGT2B4 | 1.416467303 | 0.038852503 |
| ASS1 | 1.405159694 | 0.008297489 |
| PAH | 1.400833218 | 0.046233162 |
| HDC | 1.398294263 | 0.012554531 |
| ALAD | 1.397702484 | 0.010990481 |
| OGDH | 1.395335064 | 0.011986644 |
| PTGS1 | 1.391665609 | 0.011545313 |
| ALDH1A3 | 1.390363583 | 0.011929073 |
| HK2 | 1.388619177 | 0.049786661 |
| CHAT | 1.377579711 | 0.014837379 |
| HMGCS2 | 1.371069401 | 0.021014768 |
| ALAS1 | 1.366571363 | 0.022490665 |
| GPD2 | 1.34846091 | 0.029754722 |
| TAT | 1.348105777 | 0.023837972 |
| OGDHL | 1.346330305 | 0.023661839 |
| OGDHL | 1.326680984 | 0.045600878 |
| GAD2 | 1.30928076 | 0.041858735 |
| SOAT2 | 1.301113303 | 0.047209076 |
| TYMP | 0.691630693 | 0.041589941 |
| SPTLC2 | 0.677663137 | 0.034743487 |
| HSD3B2 | 0.675769246 | 0.032459094 |
| PTGS1 | 0.666773189 | 0.03058934 |
| RRM2B | 0.656119998 | 0.025052404 |
| LIPE | 0.654581167 | 0.023090732 |
| HMGCS2 | 0.644756537 | 0.019772733 |
| ACO1 | 0.63280127 | 0.016888863 |
| G6PD | 0.611849952 | 0.010887864 |
| HSD17B6 | 0.606404935 | 0.010535183 |
| ADK | 0.586153854 | 0.036489196 |
| MYLK3 | 0.580779687 | 0.034442685 |
| SOAT2 | 0.580127012 | 0.00617189 |
| SQLE | 0.577522877 | 0.006154288 |
| ALDH1A3 | 0.577167792 | 0.005790719 |
| LTA4H | 0.575984103 | 0.006170746 |
| ALDH2 | 0.572107673 | 0.030923782 |
| IMPDH1 | 0.569592162 | 0.005215595 |
| NAT1 | 0.56769825 | 0.005166155 |
| TPH1 | 0.562214279 | 0.027398646 |
| ALDH2 | 0.557939346 | 0.025985274 |
| MYLK2 | 0.553175858 | 0.025313284 |
| FBP1 | 0.552076578 | 0.026329675 |
| LTA4H | 0.540591712 | 0.002995415 |
| DLD | 0.539984649 | 0.0210914 |
| ALDH1B1 | 0.52740414 | 0.018576582 |
| DCK | 0.527281981 | 0.017672159 |
| DCK | 0.527037711 | 0.017736797 |
| GPD2 | 0.522954741 | 0.002538286 |
| TYMP | 0.518219964 | 0.001869707 |

| | | |
|---|---|---|
| PAH | 0.503830931 | 0.013575365 |
| ALOX5 | 0.503542202 | 0.001861902 |
| PCK2 | 0.502609554 | 0.013208263 |
| ALDH3A1 | 0.500899578 | 0.013199087 |
| COX6A1 | 0.479631627 | 0.000837111 |
| HSD3B1 | 0.460100746 | 0.000564636 |
| STS | 0.450211121 | 0.006654817 |
| ADK | 0.442149818 | 0.005871307 |
| GPD2 | 0.426365527 | 0.000295298 |
| DTYMK | 0.419675922 | 0.004590806 |
| HK2 | 0.409904633 | 0.003801342 |
| SAT2 | 0.399278391 | 0.003101997 |
| ASS1 | 0.398430418 | 0.000146375 |
| RDH5 | 0.380224421 | 0.002390196 |
| MYLK2 | 0.341261484 | 0.001422072 |
| ADK | 0.313291254 | 0.000857144 |
| PCK1 | 0.246235931 | 0.000306633 |
| DLST | 0.694945044 | 0.050598169 |
| ALDH3A1 | 0.613146999 | 0.050796665 |
| COX5B | 0.611314885 | 0.051616244 |
| COX6C | 0.70690032 | 0.052306107 |
| RRM1 | 1.293774336 | 0.052948541 |
| TYR | 1.380680014 | 0.053623234 |
| SAT1 | 1.379336446 | 0.054410611 |
| TPH1 | 0.621696861 | 0.054999659 |
| PIK3C3 | 0.621208295 | 0.055194487 |
| GAD1 | 1.288092679 | 0.057005308 |
| COX6C | 0.70701871 | 0.059034094 |
| G6PC2 | 0.629880302 | 0.060588193 |
| COX4I1 | 0.705598256 | 0.061313386 |
| HSD3B2 | 0.724300562 | 0.068008422 |
| ACADL | 1.278031327 | 0.069470784 |
| ACO2 | 0.719092366 | 0.0700047 |
| COX4I1 | 1.273651608 | 0.070022998 |
| SAT2 | 0.641239366 | 0.070104113 |
| DPYD | 0.643804337 | 0.070826112 |
| PLAU | 0.638430154 | 0.071631987 |
| REN | 1.353320457 | 0.072840212 |
| DPYD | 1.356129734 | 0.073783967 |
| GAD2 | 1.27601899 | 0.074304309 |
| UCK1 | 1.361503886 | 0.075287105 |
| COX6A1 | 0.730692498 | 0.078940287 |
| ACO1 | 1.276729239 | 0.08212644 |
| ALDH7A1 | 0.658705545 | 0.085869691 |
| FBP2 | 1.346969142 | 0.086059318 |
| F2 | 0.661636899 | 0.086306976 |
| ALDH3A1 | 0.664324027 | 0.09152965 |
| ADH1A | 0.743121286 | 0.092936925 |
| SCD5 | 0.746790717 | 0.09331127 |
| UCK2 | 0.665178987 | 0.093497909 |
| LIPE | 0.744660067 | 0.095421799 |
| FBP1 | 0.670919602 | 0.096806919 |
| UCK2 | 0.655896281 | 0.097013216 |
| RRM2 | 1.254594202 | 0.099755397 |
| UCK1 | 0.677026636 | 0.101002634 |
| XDH | 1.256488093 | 0.102393691 |
| PKM | 1.337808577 | 0.103345959 |
| OGDHL | 0.755431684 | 0.103533787 |

| | | |
|---|---|---|
| ALOX5 | 0.752709195 | 0.104187448 |
| HK3 | 0.678614471 | 0.104920318 |
| ADH1A | 0.749513204 | 0.106165351 |
| PTGS2 | 0.683377996 | 0.106856809 |
| ALDH9A1 | 1.318510354 | 0.107501073 |
| ALDH1A1 | 0.759456217 | 0.111754958 |
| DTYMK | 0.68863003 | 0.116988049 |
| RRM2 | 1.234116337 | 0.11818463 |
| TYR | 0.6924164 | 0.119159508 |
| UGDH | 0.759929664 | 0.1191628 |
| COX6C | 0.7645461 | 0.120310159 |
| ADH7 | 1.314968281 | 0.121539655 |
| DDC | 0.767031855 | 0.121683502 |
| PLAT | 0.697546307 | 0.12305994 |
| COX6A1 | 0.768452238 | 0.125978171 |
| PKLR | 0.696202741 | 0.12692533 |
| APRT | 0.703042641 | 0.129139351 |
| SOAT1 | 0.772358471 | 0.131668534 |
| FBP1 | 0.700966256 | 0.13228771 |
| IMPDH1 | 1.228316289 | 0.134787401 |
| PCK2 | 1.289196521 | 0.14456012 |
| ALDH9A1 | 1.28528803 | 0.14749551 |
| G6PC | 0.713913209 | 0.149959437 |
| GNE | 0.721608081 | 0.153494246 |
| ALDH1A1 | 1.220385548 | 0.157108 |
| COX6A1 | 1.221805974 | 0.15873321 |
| UCK2 | 0.715134594 | 0.165020539 |
| MYLK2 | 0.724417297 | 0.165208847 |
| ALAS2 | 1.206536366 | 0.171167339 |
| DLD | 1.265745452 | 0.174272917 |
| PTGS1 | 0.799938448 | 0.180033863 |
| GNE | 1.264890502 | 0.183306346 |
| ADH7 | 1.265745452 | 0.183932977 |
| SQLE | 0.802779288 | 0.186905121 |
| NAT2 | 0.802542556 | 0.191314946 |
| DDC | 1.204524051 | 0.196992413 |
| PCK1 | 1.250233617 | 0.203304598 |
| REN | 0.75067758 | 0.203813572 |
| LIPE | 1.197777054 | 0.204555895 |
| ADH7 | 0.760326721 | 0.220294675 |
| ASS1 | 0.820179563 | 0.227312752 |
| GPAM | 1.187005401 | 0.228933686 |
| SOAT2 | 1.18428294 | 0.236726076 |
| ACO2 | 0.809289622 | 0.237720481 |
| HSD3B2 | 0.823967364 | 0.240098695 |
| REN | 0.766678007 | 0.242832102 |
| PYGB | 0.824204131 | 0.245469535 |
| PLAT | 1.230202489 | 0.245785524 |
| BACE1 | 0.769609386 | 0.248323774 |
| GNE | 0.766067329 | 0.24943644 |
| ALDH1A2 | 0.829530712 | 0.259952142 |
| ADK | 0.779624968 | 0.260204618 |
| OGDH | 1.164278582 | 0.269828923 |
| TYMP | 0.833792038 | 0.271208002 |
| ALDH7A1 | 0.784266292 | 0.271751469 |
| TK1 | 0.786464854 | 0.274352612 |
| SOAT1 | 0.833555289 | 0.276511883 |
| HK1 | 0.791228337 | 0.284579951 |

| | | |
|---|---|---|
| PIK3C3 | 0.79232762 | 0.286125457 |
| SAT1 | 1.164633643 | 0.287630476 |
| BACE1 | 1.211148529 | 0.292499077 |
| LPCAT2 | 1.201499377 | 0.299161774 |
| ACO1 | 1.155874368 | 0.299298817 |
| RDH5 | 0.795625378 | 0.304123724 |
| MYLK2 | 1.198934464 | 0.305137933 |
| HSD17B6 | 0.846575886 | 0.305329594 |
| PLAU | 1.206995753 | 0.308170171 |
| PIK3C3 | 0.795869669 | 0.309775524 |
| ADH1A | 0.849535123 | 0.312155003 |
| PYGL | 1.196858087 | 0.312740409 |
| G6PD | 1.150192631 | 0.313421944 |
| ALDH1A2 | 1.152204901 | 0.313517437 |
| DDC | 1.151021195 | 0.313800405 |
| SPTLC1 | 0.84906164 | 0.314016306 |
| RDH5 | 1.202110069 | 0.314178672 |
| G6PC | 1.193682372 | 0.318199505 |
| TAT | 0.847759587 | 0.318330389 |
| SCD | 1.193682372 | 0.320135842 |
| LPCAT2 | 1.191361712 | 0.327366549 |
| PLAT | 0.808450193 | 0.328945538 |
| STS | 0.807961638 | 0.330948258 |
| ALAD | 1.146996668 | 0.331010142 |
| LTA4H | 0.856282116 | 0.333684013 |
| STS | 0.813702258 | 0.339131398 |
| ALDH1A3 | 1.147588487 | 0.343220643 |
| GAD2 | 1.144037466 | 0.345089861 |
| FBP2 | 0.81541223 | 0.345792365 |
| ALDH1A2 | 1.141906795 | 0.347988186 |
| COX6B1 | 1.189529623 | 0.349344411 |
| PYGM | 0.86137204 | 0.353618844 |
| F2 | 1.179514047 | 0.354027827 |
| ALDH3A1 | 1.179025483 | 0.365604809 |
| SOAT1 | 0.859478109 | 0.370372387 |
| HK2 | 1.175971992 | 0.371769365 |
| DCK | 0.824694905 | 0.374307044 |
| RRM1 | 1.134212845 | 0.37622253 |
| RRM2B | 0.87024969 | 0.385333618 |
| ALAS2 | 0.862674071 | 0.38969479 |
| DPYD | 0.833855498 | 0.391422062 |
| PYGB | 0.873327273 | 0.394773871 |
| UGT2B4 | 0.835565482 | 0.396292725 |
| STS | 0.835931887 | 0.399050721 |
| APRT | 0.834221905 | 0.399205669 |
| IMPDH1 | 1.131371944 | 0.39953028 |
| HK1 | 1.163147211 | 0.411840166 |
| SQLE | 0.876878341 | 0.416570873 |
| ALAS1 | 1.124861673 | 0.422213582 |
| ALAS1 | 0.880074341 | 0.423066756 |
| ASS1 | 0.879719258 | 0.424858391 |
| DLD | 0.845825289 | 0.431303021 |
| TPH2 | 0.847413137 | 0.433236661 |
| G6PC | 1.165712161 | 0.433825524 |
| G6PC2 | 1.158139388 | 0.433841407 |
| IMPDH1 | 1.122849367 | 0.436421261 |
| APRT | 0.845703175 | 0.442389591 |
| COX6B1 | 0.847290971 | 0.442463754 |

| | | |
|---|---|---|
| HSD3B2 | 1.112788025 | 0.448403197 |
| CHAT | 0.885874417 | 0.449909641 |
| F2 | 1.147513161 | 0.450503324 |
| RRM1 | 1.112432886 | 0.452978928 |
| UGDH | 1.10900018 | 0.468637999 |
| HK3 | 1.136276231 | 0.482403 |
| PIK3C3 | 1.138352603 | 0.493859031 |
| F2 | 0.866222829 | 0.49403043 |
| ALAS2 | 0.895699062 | 0.496975981 |
| FBP2 | 0.863902138 | 0.498436227 |
| PYGL | 0.897119515 | 0.499608364 |
| COX6C | 0.898184792 | 0.504995723 |
| NAT1 | 0.899841993 | 0.50793741 |
| LPCAT2 | 0.867444223 | 0.51382449 |
| ALAS1 | 1.096334696 | 0.520232883 |
| CHAT | 1.099767379 | 0.537436923 |
| HK1 | 0.881123958 | 0.539340955 |
| DTYMK | 1.116611485 | 0.547351358 |
| PKLR | 0.881246117 | 0.549624774 |
| PLAT | 0.883200381 | 0.552488573 |
| HK1 | 0.881490423 | 0.556909723 |
| PYGB | 0.909074793 | 0.560951999 |
| RRM2 | 0.918307542 | 0.581544162 |
| HMGCS2 | 0.919136159 | 0.585224771 |
| IMPDH2 | 1.106229561 | 0.587901814 |
| TYMP | 1.080236499 | 0.596566964 |
| DLST | 0.923397416 | 0.612971948 |
| FBP1 | 1.097801869 | 0.616091901 |
| ACO1 | 1.073844577 | 0.626721653 |
| ALDH7A1 | 0.907506386 | 0.638538959 |
| MYLK | 0.909460636 | 0.640964682 |
| PYGL | 1.06899144 | 0.651565679 |
| IMPDH2 | 0.910559943 | 0.656888411 |
| PYGL | 0.933577179 | 0.6617484 |
| GAD2 | 0.934997621 | 0.66247867 |
| ALOX5 | 1.066032188 | 0.662709353 |
| HMGCR | 0.913491304 | 0.663034459 |
| G6PC2 | 1.083877791 | 0.666089212 |
| PTGS2 | 1.08277854 | 0.671240728 |
| HDC | 0.93677313 | 0.677272903 |
| HSD3B1 | 0.936418018 | 0.677851092 |
| ACO2 | 1.065085243 | 0.678016402 |
| HK3 | 0.920331173 | 0.684162081 |
| UCK1 | 1.07825933 | 0.686921801 |
| OGDH | 1.058930056 | 0.690467911 |
| TAT | 0.940679324 | 0.693147083 |
| OGDH | 1.059758619 | 0.698609038 |
| MYLK3 | 1.071175152 | 0.719192583 |
| MYLK | 0.93144604 | 0.724974782 |
| DLST | 1.051117681 | 0.735752252 |
| RRM2B | 0.949201904 | 0.738427735 |
| PAH | 0.931812442 | 0.739443848 |
| UGDH | 1.049223772 | 0.740797657 |
| HDC | 0.948965142 | 0.745014285 |
| HSD17B6 | 1.04531764 | 0.759798969 |
| UGT2B4 | 1.059327442 | 0.760836782 |
| PCK1 | 1.056518259 | 0.774810861 |
| XDH | 0.956777521 | 0.77502605 |

| | | |
|---|---|---|
| TPH1 | 1.055052507 | 0.776093954 |
| TAT | 0.959855141 | 0.788680131 |
| GAD1 | 1.039044037 | 0.792007875 |
| SPTLC2 | 1.040227728 | 0.793602633 |
| PCK1 | 0.950133539 | 0.799500059 |
| ACO2 | 0.962695999 | 0.804930901 |
| PYGM | 0.963998028 | 0.808272867 |
| SAT1 | 1.035848059 | 0.810227538 |
| COX4I1 | 0.965063381 | 0.821137904 |
| BACE1 | 0.954774936 | 0.821443505 |
| PYGB | 0.967312401 | 0.829527905 |
| HMGCR | 0.959171967 | 0.832686646 |
| COX5B | 1.039418542 | 0.839924791 |
| APRT | 1.041494918 | 0.841853845 |
| HK3 | 0.962836207 | 0.847873184 |
| HMGCS2 | 0.97204715 | 0.852898401 |
| LPCAT2 | 1.03551001 | 0.854651187 |
| IMPDH2 | 0.96588973 | 0.859521808 |
| SAT2 | 0.966378324 | 0.86256295 |
| UGDH | 1.026141795 | 0.862894896 |
| ACADL | 1.026260157 | 0.866113484 |
| UCKL1 | 1.030502211 | 0.874287562 |
| SCD5 | 1.019394801 | 0.897118267 |
| ACSL1 | 0.982226874 | 0.904517555 |
| COX6B1 | 1.02060881 | 0.915664759 |
| RRM2B | 0.988026951 | 0.936102058 |
| PKM | 1.014135341 | 0.941940998 |
| G6PD | 0.989210666 | 0.941953361 |
| UCK1 | 0.986042954 | 0.942989159 |
| G6PC | 1.013768962 | 0.944095223 |
| MYLK3 | 0.986775841 | 0.945472061 |
| ACADL | 1.008623189 | 0.956520099 |
| ALDH3A2 | 1.010593286 | 0.956657102 |
| UGT2B4 | 0.992027899 | 0.967237888 |
| COX5B | 0.993127121 | 0.972687198 |
| COX4I1 | 0.997141396 | 0.985388524 |
| TK1 | 0.998257077 | 0.992834591 |
| PCK2 | 0.998867762 | 0.995314887 |
