# Supplementary material for: HMGCS1 drives cholesterol-dependent membrane repair and shields tumor cells from lymphocyte attack
Source: Nat Commun. 2026 Jun 5;17:7204. doi: 10.1038/s41467-026-74022-y (PMC13396504; doi:10.1038/s41467-026-74022-y)
Supplement: Supplementary file 2 — Reporting Summary [file 41467_2026_74022_MOESM2_ESM.pdf]

nature portfolio

Corresponding author(s): Weiwei Yang

Last updated by author(s): May 15, 2026

Reporting Summary

Nature Portfolio wishes to improve the reproducibility of the work that we publish. This form provides structure for consistency and transparency in reporting. For further information on Nature Portfolio policies, see our Editorial Policies and the Editorial Policy Checklist.

Statistics

For all statistical analyses, confirm that the following items are present in the figure legend, table legend, main text, or Methods section.

n/a	Confirmed	
☐	☒	The exact sample size (*n*) for each experimental group/condition, given as a discrete number and unit of measurement
☐	☒	A statement on whether measurements were taken from distinct samples or whether the same sample was measured repeatedly
☐	☒	The statistical test(s) used AND whether they are one- or two-sided<br>*Only common tests should be described solely by name; describe more complex techniques in the Methods section.*
☒	☐	A description of all covariates tested
☒	☐	A description of any assumptions or corrections, such as tests of normality and adjustment for multiple comparisons
☐	☒	A full description of the statistical parameters including central tendency (e.g. means) or other basic estimates (e.g. regression coefficient) AND variation (e.g. standard deviation) or associated estimates of uncertainty (e.g. confidence intervals)
☐	☒	For null hypothesis testing, the test statistic (e.g. *F*, *t*, *r*) with confidence intervals, effect sizes, degrees of freedom and *P* value noted<br>*Give P values as exact values whenever suitable.*
☒	☐	For Bayesian analysis, information on the choice of priors and Markov chain Monte Carlo settings
☒	☐	For hierarchical and complex designs, identification of the appropriate level for tests and full reporting of outcomes
☐	☒	Estimates of effect sizes (e.g. Cohen's *d*, Pearson's *r*), indicating how they were calculated

Our web collection on statistics for biologists contains articles on many of the points above.

Software and code

Policy information about availability of computer code

Data collection	Immunoblotting data: Tanon-5200 Chemiluminescent Imaging System (version 1.0)<br>Cell Proliferation data: BioTek EON (version 1.0)<br>Cell PMDR data and T cell killing data: Kaleido (version 1.0)<br>Immunofluorescence staining data: LAS X (version 2.4)<br>Bioluminescence imaging data: Xenogen IVIS system (version 4.0)
Data analysis	Immunofluorescence staining analysis: ImageJ (version 1.52a)<br>Statistical analysis: GraphPad Prism (version 8.0)

For manuscripts utilizing custom algorithms or software that are central to the research but not yet described in published literature, software must be made available to editors and reviewers. We strongly encourage code deposition in a community repository (e.g. GitHub). See the Nature Portfolio guidelines for submitting code & software for further information.

Data

Policy information about availability of data

All manuscripts must include a data availability statement. This statement should provide the following information, where applicable:

- Accession codes, unique identifiers, or web links for publicly available datasets
- A description of any restrictions on data availability
- For clinical datasets or third party data, please ensure that the statement adheres to our policy

Source data are provided with this paper.

Research involving human participants, their data, or biological material

Policy information about studies with human participants or human data. See also policy information about sex, gender (identity/presentation), and sexual orientation and race, ethnicity and racism.

Reporting on sex and gender	Not applicable.
Reporting on race, ethnicity, or other socially relevant groupings	Not applicable.
Population characteristics	Age and gender were randomly distributed. Two cohorts of NSCLC patients were used in the study. IF analysis with anti-HMGCS1, anti-CHMP4B and anti-ATP1A1 (plasma membrane marker) antibodies and immunohistochemistry (IHC) analysis with anti-phospho-c-Jun S73 antibody was performed on tumor tissues from lung cancer patients receiving anti-PD-1-based immunotherapy (Response n=12; Non-response n=13) in Fig. 6c-e. Cholesterol content was measured in tumor tissues from lung cancer patients receiving anti-PD-1-based immunotherapy (Response n=10; Non-response n=10) in Fig. 6f.
Recruitment	Not applicable.
Ethics oversight	The use of human lung cancer tissues was approved by the institutional review board at Shanghai Chest Hospital (Shanghai, China) and complied with all relevant ethical regulations. Informed consent was obtained from all patients.

Note that full information on the approval of the study protocol must also be provided in the manuscript.

Field-specific reporting

Please select the one below that is the best fit for your research. If you are not sure, read the appropriate sections before making your selection.

☒ Life sciences ☐ Behavioural & social sciences ☐ Ecological, evolutionary & environmental sciences

For a reference copy of the document with all sections, see nature.com/documents/nr-reporting-summary-flat.pdf

Life sciences study design

All studies must disclose on these points even when the disclosure is negative.

Sample size	The chosen sample size are based on the numbers used for previous publications (PMID: 35315437; PMID: 31243371), which is most optimal to generate statistically significant results. No statistical methods were used to predetermine sample sizes.
Data exclusions	No samples or animals were excluded from the analyses.
Replication	All replicates are biological replicates obtained from biologically independent experiments. All attempts at replication were successful. The experiments number has been clearly stated in the figure legends.
Randomization	The samples/cells were randomized to be examined. The mice were randomized to put into separate groups/cages for experiments.
Blinding	For all experiements, the investigators were divided into two groups. One group were blinded to allocation during experiments and outcome assessment.

Reporting for specific materials, systems and methods

We require information from authors about some types of materials, experimental systems and methods used in many studies. Here, indicate whether each material, system or method listed is relevant to your study. If you are not sure if a list item applies to your research, read the appropriate section before selecting a response.

Materials & experimental systems

n/a	Involved in the study	
☐	☒	Antibodies
☐	☒	Eukaryotic cell lines
☒	☐	Palaeontology and archaeology
☐	☒	Animals and other organisms
☒	☐	Clinical data
☒	☐	Dual use research of concern
☒	☐	Plants

Methods

n/a	Involved in the study	
☒	☐	ChIP-seq
☒	☐	Flow cytometry
☒	☐	MRI-based neuroimaging

Antibodies

Antibodies used	Primary antibodies were used against: HMGCS1 (17643-1-AP, Proteintech Group); Flag (20543-1-AP, Proteintech Group); Actin (60008-1-lg, Proteintech Group); Tubulin (T5201, Sigma-Aldrich); SQLE (12544-1-AP, Proteintech Group); CHMP4B (13683-1-AP, Proteintech Group); CHMP4B (68811-2-Ig, Proteintech Group); ATP1A1 (14418-1-AP, Proteintech Group); CDH1 (20874-1-AP, Proteintech Group); c-Jun (9165T, Cell Signaling Technology); c-Jun pS73 (3270T, Cell Signaling Technology); HMGCR (ab174830, Abcam). The following secondary antibodies were used: goat-anti-mouse IgG second antibody (31160, Thermo); goat-anti-rabbit IgG second antibody (31210, Thermo); goat-anti-mouse cy3 (115-165-146, Jackson ImmunoResearch); goat-anti-rabbit Alexa Fluor 488 (A11034, Thermo). The primary antibodies were used at a 1:1000 dilution for immunoblotting and a 1:300 dilution for IF. Secondary antibodies were used at 1:3000 dilution for immunoblotting and a 1:200 dilution for IF.
Validation	All the antibodies were validated by manufacturers for indicated species and applications.

Eukaryotic cell lines

Policy information about cell lines and Sex and Gender in Research

Cell line source(s)	The human lung cancer cell lines H1299 (TCHu160) and A549 (TCHu150), the mouse Lewis lung cancer cell line (TCM 7), the human melanoma cell line A375 (TCHu155), the human colorectal cancer cell line HCT116 (TCHu 99), and the HEK293T (GNHu17) cell line were kindly provided by the Type Culture Collection of the Chinese Academy of Sciences. NK-92MI (CL-0533) cells were brought from Wuhan Pricella Biotechnology.
Authentication	Cells were authenticated using the short tandem repeat (STR) method.
Mycoplasma contamination	All cell lines were routinely tested negative for mycoplasma contamination.
Commonly misidentified lines (See ICLAC register)	No commonly misidentified cell lines were used.

Animals and other research organisms

Policy information about studies involving animals; ARRIVE guidelines recommended for reporting animal research, and Sex and Gender in Research

Laboratory animals	6-week-old female B-NDG mice were brought from Biocytogen. Six-week-old female C57BL/6 mice were purchased from SLAC.
Wild animals	The study did not involve wild animals.
Reporting on sex	All experiments were conducted with female mice to limit complications of male territorial behavior and fighting during long-term cancer experiments.
Field-collected samples	The study did not involve samples collected from the field.
Ethics oversight	All animal experiments were approved by the Institutional Animal Care and Use Committee (IACUC) of Shanghai Institute of Biochemistry and Cell Biology (approval number: SIBCB-S355-2312-40)and complied with all relevant ethical regulations.

Note that full information on the approval of the study protocol must also be provided in the manuscript.

Plants

Seed stocks

Report on the source of all seed stocks or other plant material used. If applicable, state the seed stock centre and catalogue number. If plant specimens were collected from the field, describe the collection location, date and sampling procedures.

Novel plant genotypes

Describe the methods by which all novel plant genotypes were produced. This includes those generated by transgenic approaches, gene editing, chemical/radiation-based mutagenesis and hybridization. For transgenic lines, describe the transformation method, the number of independent lines analyzed and the generation upon which experiments were performed. For gene-edited lines, describe the editor used, the endogenous sequence targeted for editing, the targeting guide RNA sequence (if applicable) and how the editor was applied.

Authentication

Describe any authentication procedures for each seed stock used or novel genotype generated. Describe any experiments used to assess the effect of a mutation and, where applicable, how potential secondary effects (e.g. second site T-DNA insertions, mosiacism, off-target gene editing) were examined.
