# Supplementary material for: HMGCS1 drives cholesterol-dependent membrane repair and shields tumor cells from lymphocyte attack
Source: Nat Commun. 2026 Jun 5;17:7204. doi: 10.1038/s41467-026-74022-y (PMC13396504; doi:10.1038/s41467-026-74022-y)
Supplement: Supplementary file 3 — Transparent Peer Review file [file 41467_2026_74022_MOESM3_ESM.pdf]

nature portfolio

Peer Review File

HMGCS1 drives cholesterol-dependent membrane repair and shields tumor cells from lymphocyte attack

Corresponding Author: Dr Weiwei Yang

This file contains all reviewer reports in order by version, followed by all author rebuttals in order by version.

Attachments originally included by the reviewers as part of their assessment can be found at the end of this file.

Version 0:

Reviewer comments:

Reviewer #1

(Remarks to the Author)
In this manuscript, the authors identified HMGCS1, a key enzyme in cholesterol biosynthesis, as critical regulators of perforin-induced plasma membrane (PM) damage, through a siRNA screen approach aimed at 111 metabolic enzymes. They reveal a new tumor evasion mechanism wherein HMGCS1 facilitates cholesterol-dependent PM repair through activating the de novo cholesterol synthesis. The experiments were performed well and the conclusions drawn from the data were well-founded. However, several aspects need to be enhanced before publication.

(1) HMGCS1 is not the rate-limiting enzyme in cholesterol biosynthesis, and the results in Figure 2I-2J indicate that depleting SQLE or inhibiting the activity of HMGCR also increasing SLO-induced PM damage rate. Whether Double or triple KO/KD of these three enzymes can further aggravate SLO-induced PM damage? Why SQLE and HMGCR is not hit from the siRNA screen?

(2) In Figure S3A, the immunoblotting analyses of HMGCS1-depleted A549 cell suggest that CHMP4B protein expression level is greatly decreased, while another protein CDH1 is also slightly decreased. The authors should clarify the purpose to detect CDH1, and explain whether CDH1 also play a role in HMGCS1 induced PM repair process. Besides CHMP4B, whether other ESCART components also contribute to the HMGCS1 induced cholesterol dependent PM repair process?

(3) In Figure 4, in the in vitro and in vivo experiment to verify HMGCS1 function in CTLs-mediated PM damage, the authors only used HMGCS1 depleted tumor cells. With wild-type tumor cells, whether adding HMGCS1 inhibitor to the co-culture system or treating with HMGCS1 inhibitor and CAR-T cells simultaneously in the animal model would have benefit?

(4) There are no representative IHC plots in Figure 6C and the cohort is limited. Larger sample size would be more convincing.

(5) Figure S5E, the increased expression of HMGCS1 in c-Jun overexpressed A549 cells is not obvious, quantification of multiple independent experiment would be more convincing.

Reviewer #2

(Remarks to the Author)
In this study, the authors elucidate a unique mechanism through which HMGCS1 promotes plasma membrane repair via cholesterol synthesis: cholesterol not only acts as a signaling molecule to facilitate ESCRT-mediated membrane repair but also serves as a structural component of the membrane. Furthermore, they found that oncogenic mutations, cytokine signaling, and hypoxia activate c-Jun to upregulate HMGCS1 expression, thereby enhancing tumor cell resistance to immune cell killing. The role of HMGCS1 in promoting plasma membrane repair is critical for tumor cell resistance to PD-1 inhibitors, CAR-T, or NK cell-mediated immunotherapies, underscoring the potential of targeting HMGCS1 to improve the efficacy of immunotherapies. Thus, this study demonstrates considerable innovation, supported by extensive

experimentation and rigorous logical reasoning. However, the following questions may still warrant consideration.

1.Among the significantly different candidate metabolic enzymes shown in Figure 1B, what distinguishes HMGCS1 and led to its selection as the focus of this research?

2.Could cholesterol present in the cell culture medium interfere with the experimental results? Would the outcomes of SLO treatment differ between shHMGCS1 cell lines cultured in medium with or without cholesterol supplementation?

3.Is there any change in the levels of the HMGCS1 product HMG-CoA in Figure 2D? There are lots of intermediate metabolites from cholesterol synthesis. Alteration of HMGCS1 expression also affect the levels of these intermediate metabolites. Do these these intermediate metabolites affect the ESCRT-mediated membrane repair and tumor cell resistance to lymphocyte killing?

4.HMGCS1 overexpression will produce Geranylgeranyl diphosphate (GGPP) and farnesyl pyrophosphate (FPP), which can be used as geranylgeranylation and farnesylation substrates in protein prenylation. Protein prenylation is one of the most important function of the mevalonate anabolic pathway. Are protein prenylation involved in ESCRT-mediated membrane repair and tumor cell resistance to lymphocyte killing?

5.Whether membrane localization of CHMP4B is necessary for Cholesterol supports PM repair and tumor cell resistance to lymphocyte killing?

6.As noted by the authors in line 251, c-Jun plays multiple roles within complex regulatory networks, where it can engage in crosstalk, amplify, and integrate various signals involved in tissue development and disease. In lines 240–250, the authors overexpress or knock down c-Jun—how do these manipulations affect tumor cell resistance to lymphocyte killing?

7.In lines 333–335, the authors state that "HMGCS1 is not a rate-limiting enzyme in cholesterol synthesis, and targeting HMGCS1 may have fewer side effects than targeting HMGCR." What is the basis for this claim? Is it because HMGCS1 is more significantly upregulated in human tumors compared to HMGCR?

8.Clinical revelance: Whether the contents of the moleculeus in the pathway of c-Jun-HMGCS1- cholesterol- CHMP4B are altered in clinical tumors resistance to immune therapy.

Reviewer #3

(Remarks to the Author)
Review of “HMGCS1 drives cholesterol-dependent membrane repair and shields tumor cells from lymphocyte attack”

Summary to place the manuscript in the context of the field

Cancer cells employ diverse mechanisms to avoid detection and destruction by the immune system. Many of these can reduce the effectiveness of various immunotherapies in patients, including downregulation of antigen presentation machinery, upregulation of immune checkpoint molecules, and secretion of immunosuppressive cytokines. By uncovering and characterizing the array of immune evasion mechanisms that tumor cells employ, the field can work to develop more effective and personalized immunotherapies to treat patients. The study by Zhang et al. seeks to understand how lung cancer cells harness metabolic programs to reduce membrane damage caused by perforin, a pore-forming protein and critical mediator of Granzyme B-induced programmed cell death. Through an imaging-based siRNA screen of metabolic enzymes, Zhang et al. uncover the cholesterol synthesis gene HMGCS1 as a key player in maintaining membrane integrity after pore formation. Zhang et al. employed a series of in vitro and in vivo assays to uncover the mechanism by which HMCGS1 mediates immune evasion and the resulting effects on immunotherapy efficacy.

Summary of the study
Zhang et al. sought to identify key metabolic enzymes involved in plasma membrane (PM) repair, hypothesizing that these enzymes might contribute to resistance to lymphocyte killing. They used a library of 111 siRNAs to screen key metabolic enzymes in A549 cells following Streptolysin O (SLO, a perforin mimetic) treatment. Through this screen, they identified depletion of HMGCS1, a key cholesterol synthesis enzyme, as a significant enhancer of PM damage. They validated this using shRNAs, and by rescuing with HMGCS1-resistant mutants. Overexpression also showed reduced PM damage after SLO treatment. The authors determined that HMGCS1 is essential for membrane repair to rescue cell viability following SLO treatment.

To determine if the enzymatic activity of HMGSC1 is essential for membrane repair, Zhang et al. used an enzymatically dead variant to show that it is unable to repair membrane damage following SLO treatment. They also demonstrated that HMGSC1 depletion significantly decreases cholesterol levels in the cell and cell membrane, mechanistically linking the role of HMGSC1 in cholesterol synthesis to membrane repair. They orthogonally validated these findings by targeting additional key cholesterol-synthesis genes and providing exogenous, labeled cholesterol molecules to rescue membrane repair. The authors also identify CHMP4B localization as a key mediator of HMGSC1-dependent membrane repair.

Using several cell lines and in vivo models of tumor-immune killing, the authors implicated HMGCS1 activity in resistance to

immune killing via membrane repair. Notably, the authors found that exposure to NK cells drives upregulation of HMGSC1 in tumor cells. The authors probed the TNF-alpha signaling pathway using small-molecule inhibitors, identifying the transcription factor c-Jun as a key driver of HMGCS1 expression. They validated this using ChIP and overexpression experiments. Importantly, they showed that c-Jun depletion partially phenocopied HMGCS1 loss in a CAR-T co-culture assay. Further, they reported that expression of common oncogenic driver mutations known to activate c-Jun increased HMGCS1 mutation in H1299 cells.

Finally, the authors explored RNA-seq data from the GEO database to examine how HMGCS1 levels relate to immunotherapy efficacy. They determined that the PD1 non-responders in melanoma have elevated levels of HMGCS1 RNA. Single-cell data from colorectal cancer (CRC) patients and IF analysis in lung cancer samples showed a similar trend. By stratifying patients into HMGCS1 high or low groups, they found that high levels of HMGCS1 are associated with significantly worse patient prognosis. Taken together, the authors argue that HMGCS1 could be a predictive biomarker of immunotherapy efficacy.

Major points
1. Zhang et al. perform all in vitro and in vivo studies of HMCGS1 using A549 and H1299 cells, two models of human lung cancer. However, in Figure 6, they perform analysis of immunotherapy response in other cancer types, including melanoma and colorectal cancer. From these analyses, they suggest that high HMCGS1 expression may be a more universal immune evasion mechanism. This argument would be substantially strengthened by performing their PMDR assay cells derived from different cancer types, particularly melanoma and CRC. Validation in at least one cell line per tumor type would suffice.
2. In their in vivo studies, Zhang et al. demonstrate that knockdown of HMCGS1 leads to significantly smaller subcutaneous tumors in immunodeficient mice treated by transplantation of NK or CAR-T cells, as well as immunocompetent mice treated with anti-PD-1. While these data are promising, additional pieces of data are requested, as described below.
a. Despite showing varying volumes at similar timepoints, all in vivo experiments appear to end at day 17-19. Survival studies should be conducted to determine whether immunotherapies significantly prolong survival in HMCGS1 depleted cohorts.
b. Zhang et al. argue that tumor microenvironment can play a role in elevating HMCGS1 expression in cancer cells. Orthotopic in vivo tumor burden and survival studies in immunocompetent mice with the LLC cancer model with or without immunotherapy are essential in arguing this point.
3. To study the role of the ESCRT complex in promoting membrane repair, the authors focus on the localization of the protein CHMP4B once bound to cholesterol. Through mutagenesis studies, they identify cholesterol recognition motif 2 as a key mediator in binding cholesterol and promoting plasma membrane repair. Analysis of the other core members of the ESCRT complex (e.g. CHMP2, CHMP3, CHMP6) for cholesterol binding motifs would further strengthen this point and potentially identify other proteins that could be targeted therapeutically. However, no formal experimental validation of these other possible motifs is requested by this reviewer, but analysis and discussion are recommended.

Minor points
1. Citations for foundational papers identifying the mechanism of perforin pore formation, such as Keefe et al, Immunity (2005), should be included.

Version 1:

Reviewer comments:

Reviewer #1

(Remarks to the Author)
The authors clearly addressed the reviewer's questions and concerns. There is no further comments at this moment.

Reviewer #2

(Remarks to the Author)
The authors have conducted extensive experiments to address all my comments, and the manuscript is now suitable for publication.

Reviewer #3

(Remarks to the Author)
The authors have adequately addressed our comments and concerns. The new results are informative and further clarify and support the main findings.

FYI: Fig. 5f panel has a typo. It should be "promoter" instead of "promotor".

Open Access This Peer Review File is licensed under a Creative Commons Attribution 4.0 International License, which permits use, sharing, adaptation, distribution and reproduction in any medium or format, as long as you give appropriate credit to the original author(s) and the source, provide a link to the Creative Commons license, and indicate if changes were made.
In cases where reviewers are anonymous, credit should be given to 'Anonymous Referee' and the source.
The images or other third party material in this Peer Review File are included in the article’s Creative Commons license, unless indicated otherwise in a credit line to the material. If material is not included in the article’s Creative Commons license and your intended use is not permitted by statutory regulation or exceeds the permitted use, you will need to obtain permission directly from the copyright holder.
To view a copy of this license, visit https://creativecommons.org/licenses/by/4.0/

Reviewer #1 (Remarks to the Author):

In this manuscript, the authors identified HMGCS1, a key enzyme in cholesterol biosynthesis, as critical regulators of perforin-induced plasma membrane (PM) damage, through a siRNA screen approach aimed at 111 metabolic enzymes. They reveal a new tumor evasion mechanism wherein HMGCS1 facilitates cholesterol-dependent PM repair through activating the de novo cholesterol synthesis. The experiments were performed well and the conclusions drawn from the data were well-founded. However, several aspects need to be enhanced before publication.

Response: Thank you for the constructive comments and insightful suggestions. We have conducted additional experiments to address the concerns raised. All changes we have made in the manuscript have been highlighted in yellow.

Point 1. HMGCS1 is not the rate-limiting enzyme in cholesterol biosynthesis, and the results in Figure 2I-2J indicate that depleting SQLE or inhibiting the activity of HMGCR also increasing SLO-induced PM damage rate. Whether Double or triple KO/KD of these three enzymes can further aggravate SLO-induced PM damage? Why SQLE and HMGCR is not hit from the siRNA screen?

Response: Thank you for the insightful suggestion. As suggested by the reviewer, we generated a triple knockdown (KD) of HMGCS1, HMGCR, and SQLE in A549 cells. As shown in Supplementary Fig. 2e, compared to HMGCS1 KD, the triple KD further aggravated SLO-induced PM damage. The results were described in line 165.

In addition, we have included the full siRNA screening result as Supplementary Table 2. Both SQLE and HMGCR were indeed identified as hits in the screening.

Point 2. In Figure S3A, the immunoblotting analyses of HMGCS1-depleted A549 cell suggest that CHMP4B protein expression level is greatly decreased, while another protein CDH1 is also slightly decreased. The authors should clarify the purpose to detect CDH1, and explain whether CDH1 also play a role in HMGCS1 induced PM repair process. Besides CHMP4B, whether other ESCART components also contribute to the HMGCS1 induced cholesterol dependent PM repair process?

Response: We apologize for the confusion. In Figure S3A, CDH1 (E-cadherin) was used as a loading control for membrane proteins. To avoid the confusion, we have repeated the experiment to replace CDH1 with the well-established PM marker ATP1A1 as the loading control. The result confirmed that HMGCS1 depletion significantly reduces the PM localization of CHMP4B (Supplementary Fig. 3a). The figure legend has been updated to clarify the use of ATP1A1 as a membrane protein loading control.

To examine whether other ESCRT components contribute to the HMGCS1-induced, cholesterol-dependent PM repair pathway, we depleted CHMP2B, another ESCRT component, in H1299 cells stably expressing either shNT or shHMGCS1. As shown in Supplementary Fig. 3g, under SLO treatment, HMGCS1 depletion impaired PM integrity, whereas cholesterol supplementation restored PM integrity in HMGCS1-depleted cells expressing control siRNA (siNT). In contrast, this cholesterol-mediated recovery was completely abolished in CHMP2B-knockdown (siCHMP2B) cells (Supplementary Fig. 3g). The knockdown efficiency of CHMP2B siRNA was validated in Supplementary Fig. 3h. These results indicate that multiple ESCRT components, including CHMP2B, participate in the cholesterol-dependent PM

repair process regulated by HMGCS1. The results were described in line 220.

Point 3. In Figure 4, in the in vitro and in vivo experiment to verify HMGCS1 function in CTLs-mediated PM damage, the authors only used HMGCS1 depleted tumor cells. With wild-type tumor cells, whether adding HMGCS1 inhibitor to the co-culture system or treating with HMGCS1 inhibitor and CAR-T cells simultaneously in the animal model would have benefit?

Response: Thank you for this insightful suggestion. To address this point, we directly added the HMGCS1 inhibitor hymeglusin to co-culture systems of A549-CD19 or H1299-CD19 cells with CD19-targeting CAR-T cells. As shown in Supplementary Fig. 4c, hymeglusin treatment enhanced CAR-T cell-mediated cytotoxicity against tumor cells, suggesting the translational potential of targeting HMGCS1 in combination with CAR-T therapy. The results were described in line 249.

Unfortunately, due to the inability to secure sufficient amount of hymeglusin (about 30 mg) for *in vivo* studies, we were unable to evaluate the combined administration of hymeglusin and CAR-T cells in animal models.

Point 4. There are no representative IHC plots in Figure 6C and the cohort is limited. Larger sample size would be more convincing.

Response: This point was well taken. We have expanded the patient cohort as recommended and similar results were obtained. We have now also included representative IF images in Fig. 6c to better illustrate our findings.

Point 5. Figure S5E, the increased expression of HMGCS1 in c-Jun overexpressed A549 cells is not obvious, quantification of multiple independent experiment would be more convincing.

Response: As suggested by the reviewer, we have now performed quantitative analyses of three independent experiments with statistical comparisons, and these data have been added to the revised Fig. 5e.

Reviewer #2 (Remarks to the Author):

In this study, the authors elucidate a unique mechanism through which HMGCS1 promotes plasma membrane repair via cholesterol synthesis: cholesterol not only acts as a signaling molecule to facilitate ESCRT-mediated membrane repair but also serves as a structural component of the membrane. Furthermore, they found that oncogenic mutations, cytokine signaling, and hypoxia activate c-Jun to upregulate HMGCS1 expression, thereby enhancing tumor cell resistance to immune cell killing. The role of HMGCS1 in promoting plasma membrane repair is critical for tumor cell resistance to PD-1 inhibitors, CAR-T, or NK cell-mediated immunotherapies, underscoring the potential of targeting HMGCS1 to improve the efficacy of immunotherapies. Thus, this study demonstrates considerable innovation, supported by extensive experimentation and rigorous logical reasoning. However, the following questions may still warrant consideration.

Response: Thank you for the constructive comments and insightful suggestions. To address the concerns raised, we have conducted additional experiments and revised the discussion in the Discussion section. All changes we have made in the manuscript have been highlighted in yellow.

Point 1. Among the significantly different candidate metabolic enzymes shown in Figure 1B, what distinguishes HMGCS1 and led to its selection as the focus of this research?

Response: Thank you for the constructive comment. In our preliminary siRNA screen, we targeted 111 key metabolic enzymes. To ensure statistical robustness, the screen was designed with four independent siRNAs per gene, each performed in triplicate. A gene was considered a candidate if its knockdown increased the PMDR by >1.5-fold compared to the control ($p < 0.05$).

Among those candidates, HMGCS1 emerged as the top hit because it was the only candidate for which all four independent siRNAs significantly increased PMDR in A549 cells—representing the highest number of effective siRNAs across the entire screen. This consistent and robust phenotype formed the primary rationale for selecting HMGCS1 as the focus of subsequent investigation. We have clarified these methodological details in the revised Methods section. The description can be found in line 551.

Point 2. Could cholesterol present in the cell culture medium interfere with the experimental results? Would the outcomes of SLO treatment differ between shHMGCS1 cell lines cultured in medium with or without cholesterol supplementation?

Response: To evaluate the potential influence of extracellular cholesterol on our findings, we compared SLO-induced PMDR in HMGCS1-depleted A549 cells cultured in medium containing either normal serum (NS) or lipoprotein-deficient serum (LPDS, which is depleted of cholesterol and other lipids). As shown in Supplementary Fig. 2h, HMGCS1 knockdown resulted in a more pronounced increase in PMDR under LPDS conditions than under NS conditions, as assessed by PI and Hoechst staining. This result suggests that the availability of exogenous cholesterol can modulate the effect of HMGCS1 loss on membrane repair capacity. This result was described in line 178.

Point 3. Is there any change in the levels of the HMGCS1 product HMG-CoA in Figure 2D? There are lots of intermediate metabolites from cholesterol synthesis. Alteration of HMGCS1 expression also affect the levels of these intermediate metabolites. Do these intermediate metabolites affect the ESCRT-mediated membrane repair and tumor cell resistance to lymphocyte killing?

Response: Thank you for the insightful suggestion.

1. As suggested, we quantified HMG-CoA and confirmed that HMGCS1 knockdown in H1299 cells significantly decreased its level (Fig. 2d).

2. To further investigate whether downstream metabolites in the cholesterol synthesis contribute to ESCRT-mediated membrane repair and tumor cell resistance to lymphocyte killing, we specifically increased the concentration of individual metabolites through two approaches: (1) Overexpressing an upstream enzyme combined with knockdown of its immediate downstream enzyme to accumulate the target metabolite, and (2) Supplementing cells with a cell-permeable precursor while knocking down the enzyme responsible for its further metabolism.

Specifically, to increase HMG-CoA levels, we overexpressed HMGCS1 while knocking down HMGCR. To elevate MVA, which is permeable to cells, we supplemented exogenous MVA while knocking down MVK to block its conversion to MVA-P. For MVA-P and GPP, which cannot enter cells directly, we supplemented MVA while knocking down PMVK or FDFT1, respectively, to allow intracellular accumulation of MVA-P or GPP through blocked downstream metabolism (Supplementary Fig. 5a-c).

Importantly, none of these interventions—which effectively raised intracellular levels of HMG-CoA, MVA, MVA-P, or GPP—enhanced plasma membrane repair following SLO-induced damage, nor did they improve tumor cell resistance to lymphocyte-mediated killing (Supplementary Fig. 5d,e). The expected changes in metabolite levels were confirmed by mass spectrometry. Of note, the levels of cholesterol were not altered by these interventions (Supplementary Fig. 5f), which may be due to the addition of upstream metabolites, together with the possibly incomplete knockdown of downstream enzymes. These results are presented in line 274 of the revised manuscript.

Point 4. HMGCS1 overexpression will produce Geranylgeranyl diphosphate (GGPP) and farnesyl pyrophosphate (FPP), which can be used as geranylgeranylation and farnesylation substrates in protein prenylation. Protein prenylation is one of the most important function of the mevalonate anabolic pathway. Are protein prenylation involved in ESCRT-mediated membrane repair and tumor cell resistance to lymphocyte killing?

Response: We thank the reviewer for this insightful suggestion. To test this hypothesis, we treated HMGCS1-overexpressing A549 cells with the prenylation inhibitor L778123. As shown in Supplementary Fig. 4e, prenylation inhibition did not abrogate HMGCS1 overexpression-inhibited PMDR. Consistently, L778123 treatment also failed to diminish the enhanced resistance to lymphocyte killing conferred by HMGCS1 overexpression (Supplementary Fig. 4f). Together, these results suggest that protein prenylation is not critically involved in HMGCS1-facilitated, ESCRT-mediated membrane repair or the resulting tumor cell resistance to lymphocyte killing. The results were described in line 263.

Point 5. Whether membrane localization of CHMP4B is necessary for Cholesterol supports PM repair and tumor cell resistance to lymphocyte killing?

Response: Thank you for this constructive comment. To address this point, we performed sequence homology analysis based on a related study (PMID: 24139821) and identified phenylalanine at position 8 (F8) as a critical residue for the membrane localization of human CHMP4B. We constructed an F8E mutation and confirmed that the mutation abrogated membrane localization of CHMP4B under SLO treatment (Supplementary Fig. 3d). Next, we depleted endogenous CHMP4B in H1299 cells and rescued them with either rCHMP4B WT or F8E mutant (Supplementary Fig. 3e), followed by cholesterol pre-treatment prior to SLO challenge. As indicated in Supplementary Fig. 3f, cholesterol-mediated enhancement of PM repair was evident in cells expressing rCHMP4B-WT, but was completely abolished in cells expressing the rCHMP4B-F8E mutant. Consistently, the F8E mutant also failed to support cholesterol-enhanced resistance to lymphocyte-mediated killing (Supplementary Fig. 4d). Taken together, these results demonstrate that membrane localization of CHMP4B is essential for cholesterol-promoted plasma membrane repair and for tumor cell resistance to lymphocyte-mediated cytotoxicity, as described in line 209 and line 256.

Point 6. As noted by the authors in line 251, c-Jun plays multiple roles within complex regulatory networks, where it can engage in crosstalk, amplify, and integrate various signals involved in tissue development and disease. In lines 240–250, the authors overexpress or knock down c-Jun—how do these manipulations affect tumor cell resistance to lymphocyte killing?

Response: This point was well taken. To assess the role of c-Jun in this context, we modulated its expression in A549-CD19 and H1299-CD19 cells and co-cultured them with CD19 CAR-T cells. c-Jun overexpression enhanced tumor cell resistance to CAR-T cell killing. Conversely, knockdown of c-Jun sensitized the tumor cells to lymphocyte-mediated cytotoxicity (Supplementary Fig. 7h,i). The results were described in line 342.

Point 7. In lines 333–335, the authors state that "HMGCS1 is not a rate-limiting enzyme in cholesterol synthesis, and targeting HMGCS1 may have fewer side effects than targeting HMGCR." What is the basis for this claim? Is it because HMGCS1 is more significantly upregulated in human tumors compared to HMGCR?

Response: To address the concern, we further clarify the rationale behind the claim.

1. Our data show that HMGCS1 expression is upregulated in tumor cells by oncogenic activation or cytokine signaling (Fig. 5) and that tumors develop a specific reliance on this enzyme in the context of immune escape (Fig. 4). Consistently, HMGCS1 expression is also elevated in immune-resistant tumor tissues (Fig. 6). Indeed, HMGCS1 is more significantly upregulated than HMGCR, although HMGCR is also upregulated in these tissues (Figure below).

2. HMGCR is the classic rate-limiting enzyme of the mevalonate pathway and is tightly regulated to maintain cholesterol homeostasis in normal cells. Although HMGCR is also upregulated in these immune-resistant tumor tissues, inhibiting HMGCR is likely to cause broad metabolic disruption across cell types, potentially leading to significant systemic side effects. In contrast, HMGCS1 catalyzes a non-rate-limiting step. Targeting a non-rate-limiting

enzyme may allow for more selective effects on tumor cells, especially those that have become dependent on its activity, while minimizing impact on normal tissues.

Due to its non-rate-limiting role and its context-specific upregulation during tumor immune evasion, we propose that targeting HMGCS1 may offer a wider therapeutic window. We have revised the relevant section to reflect this reasoning more clearly and thank the reviewer for prompting this important clarification. The description can be found in line 446.

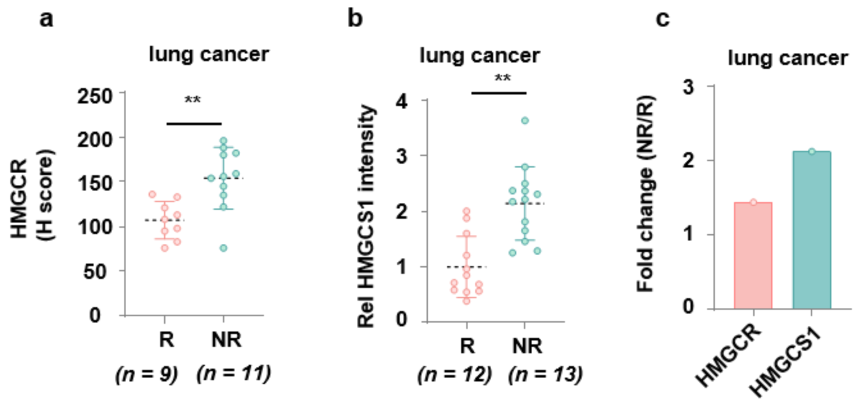

Fig. R1. HMGCS1 is more significantly upregulated than HMGCR in immune-resistant tumor tissues

a. IHC staining was performed using anti-HMGCR antibody on tumor tissues from lung cancer patients after ICB treatment (Response n = 9; Non-response n = 11). Semiquantitative scoring was carried out (two-tailed Student's t-test).

b. IF staining was performed using anti-HMGCS1 antibody on tumor tissues from lung cancer patients after ICB treatment (Response n = 12; Non-response n = 13). The average intensity of HMGCS1 in tumor tissues was calculated (two-tailed Student's t-test).

c. The fold change in HMGCR and HMGCS1 expression was calculated between immune-non-responsive and immune-responsive tumor tissues.

Point 8. Clinical revelance: Whether the contents of the moleculeus in the pathway of c-Jun-HMGCS1- cholesterol- CHMP4B are altered in clinical tumors resistance to immune therapy.

Response: This point was well taken. We assemble a cohort of tumor samples from patients who received immunotherapy. We performed IF or IHC staining on these tumor samples to directly assess whether the levels of phospho-c-Jun, HMGCS1, and plasma membrane (PM)-located CHMP4B are elevated in tumors that are resistant to immune therapy compared to those that respond. We observed that non-responders exhibited significantly higher levels of phospho-c-Jun, HMGCS1, and PM CHMP4B in their tumor tissues compared to responders (Fig. 6c–6e). Additionally, tumor cholesterol levels were also elevated in non-responding patients (Fig. 6f). The results were described in line 373.

Reviewer #3 (Remarks to the Author):

Review of "HMGCS1 drives cholesterol-dependent membrane repair and shields tumor cells from lymphocyte attack"

Summary to place the manuscript in the context of the field

Cancer cells employ diverse mechanisms to avoid detection and destruction by the immune system. Many of these can reduce the effectiveness of various immunotherapies in patients, including downregulation of antigen presentation machinery, upregulation of immune checkpoint molecules, and secretion of immunosuppressive cytokines. By uncovering and characterizing the array of immune evasion mechanisms that tumor cells employ, the field can work to develop more effective and personalized immunotherapies to treat patients. The study by Zhang et al. seeks to understand how lung cancer cells harness metabolic programs to reduce membrane damage caused by perforin, a pore-forming protein and critical mediator of Granzyme B-induced programmed cell death. Through an imaging-based siRNA screen of metabolic enzymes, Zhang et al. uncover the cholesterol synthesis gene HMGCS1 as a key player in maintaining membrane integrity after pore formation. Zhang et al. employed a series of in vitro and in vivo assays to uncover the mechanism by which HMCGS1 mediates immune evasion and the resulting effects on immunotherapy efficacy.

Response: Thank you for the constructive comments and insightful suggestions. To address the concerns raised, we have conducted additional experiments and expanded the discussion in the Discussion section. All changes we have made in the manuscript have been highlighted in yellow.

Major points

Point 1. Zhang et al. perform all in vitro and in vivo studies of HMCGS1 using A549 and H1299 cells, two models of human lung cancer. However, in Figure 6, they perform analysis of immunotherapy response in other cancer types, including melanoma and colorectal cancer. From these analyses, they suggest that high HMCGS1 expression may be a more universal immune evasion mechanism. This argument would be substantially strengthened by performing their PMDR assay cells derived from different cancer types, particularly melanoma and CRC. Validation in at least one cell line per tumor type would suffice.

Response: Thank you for this constructive suggestion. As suggested, we depleted HMGCS1 in A375 human melanoma cells line and HCT116 colorectal cancer cell line. Consistent with our prior results, PMDR assays confirmed that HMGCS1 depletion significantly sensitized both cell types to SLO-induced membrane damage (Supplementary Fig. 8a,b), supporting the broader role of HMGCS1 as a conserved mechanism of immune evasion across multiple cancer types. The results were described in line 369.

Point 2. In their in vivo studies, Zhang et al. demonstrate that knockdown of HMCGS1 leads to significantly smaller subcutaneous tumors in immunodeficient mice treated by transplantation of NK or CAR-T cells, as well as immunocompetent mice treated with anti-PD-1. While these data are promising, additional pieces of data are requested, as described below.

a. Despite showing varying volumes at similar timepoints, all in vivo experiments appear to end at day 17-19. Survival studies should be conducted to determine whether immunotherapies significantly prolong survival in HMCGS1 depleted cohorts.

Response: Thank you for this constructive comment. As suggested, we conducted additional survival analyses in relevant mouse models. Specifically, in the subcutaneous tumor model using A549 cells and B-NDG mouse, the combination of HMGCS1 knockdown with either CAR-T or NK cell treatment significantly extended overall survival compared to HMGCS1 depletion alone (Supplementary Fig. 6c,d). Similarly, in the orthotopic lung cancer model with LLC cells and C57BL/6 mouse, Hmgcs1 depletion combined with anti-PD-1 treatment also markedly improved mouse survival (Supplementary Fig. 6e). These results are described in line 299 and line 311.

b. Zhang et al. argue that tumor microenvironment can play a role in elevating HMCGS1 expression in cancer cells. Orthotopic in vivo tumor burden and survival studies in immunocompetent mice with the LLC cancer model with or without immunotherapy are essential in arguing this point.

Response: We appreciate the reviewer's insightful suggestion. Accordingly, we established an orthotopic lung cancer model by implanting luciferase-expressing LLC cells, with or without Hmgcs1 knockdown, into the left lung of C57BL/6 mice. The mice were subsequently administered either control IgG2a or anti-PD-1. Tumor progression and mouse survival were then assessed. As shown in Fig. 4j, Hmgcs1 knockdown slightly attenuated tumor growth in the control IgG2a-treated group and significantly potentiated the efficacy of anti-PD-1 therapy. Importantly, the combination of Hmgcs1 knockdown and anti-PD-1 treatment produced the most substantial survival benefit (Supplementary Fig. 6e). These results are described in line 305 and line 311.

Point 3. To study the role of the ESCRT complex in promoting membrane repair, the authors focus on the localization of the protein CHMP4B once bound to cholesterol. Through mutagenesis studies, they identify cholesterol recognition motif 2 as a key mediator in binding cholesterol and promoting plasma membrane repair. Analysis of the other core members of the ESCRT complex (e.g. CHMP2, CHMP3, CHMP6) for cholesterol binding motifs would further strengthen this point and potentially identify other proteins that could be targeted therapeutically. However, no formal experimental validation of these other possible motifs is requested by this reviewer, but analysis and discussion are recommended.

Response: Thank you for this constructive suggestion. Following the recommendation, we performed a systematic in silico analysis to examine other core ESCRT proteins for the presence of established cholesterol-recognition motifs ((L/V)-$X_{1–5}$-(Y)-$X_{1–5}$-(K/R)). Notably, our analysis indicates that many of these proteins also contain putative cholesterol-recognition motifs (Supplementary Fig. 9). We have added a paragraph to the Discussion noting that cholesterol-dependent regulation of the ESCRT machinery may not be limited to CHMP4B, and that targeting these interactions in other ESCRT components could represent a promising direction for future therapeutic development. This discussion can be found in line 424.

Minor points

Point 1. Citations for foundational papers identifying the mechanism of perforin pore formation, such as Keefe et al, Immunity (2005), should be included.

Response: Thank you for this constructive suggestion. We have now included the citation of the foundational paper in the revised manuscript.

Reviewer #3 (Remarks to the Author):

The authors have adequately addressed our comments and concerns. The new results are informative and further clarify and support the main findings.

Response: We appreciate the positive assessment and are pleased to hear that the revisions have effectively addressed the comments and further strengthened the manuscript.

FYI: Fig. 5f panel has a typo. It should be "promoter" instead of "promotor".

Response: Thank you for pointing this out. The typo in Fig. 5f has been corrected to “promoter”.

Review of "*HMGCS1 drives cholesterol-dependent membrane repair and shields tumor cells from lymphocyte attack*"

Summary to place the manuscript in the context of the field

Cancer cells employ diverse mechanisms to avoid detection and destruction by the immune system. Many of these can reduce the effectiveness of various immunotherapies in patients, including downregulation of antigen presentation machinery, upregulation of immune checkpoint molecules, and secretion of immunosuppressive cytokines. By uncovering and characterizing the array of immune evasion mechanisms that tumor cells employ, the field can work to develop more effective and personalized immunotherapies to treat patients. The study by Zhang et al. seeks to understand how lung cancer cells harness metabolic programs to reduce membrane damage caused by perforin, a pore-forming protein and critical mediator of Granzyme B-induced programmed cell death. Through an imaging-based siRNA screen of metabolic enzymes, Zhang et al. uncover the cholesterol synthesis gene *HMGCS1* as a key player in maintaining membrane integrity after pore formation. Zhang et al. employed a series of *in vitro* and *in vivo* assays to uncover the mechanism by which *HMCGS1* mediates immune evasion and the resulting effects on immunotherapy efficacy.

Summary of the study

Zhang et al. sought to identify key metabolic enzymes involved in plasma membrane (PM) repair, hypothesizing that these enzymes might contribute to resistance to lymphocyte killing. They used a library of 111 siRNAs to screen key metabolic enzymes in A549 cells following Streptolysin O (SLO, a perforin mimetic) treatment. Through this screen, they identified depletion of *HMGCS1*, a key cholesterol synthesis enzyme, as a significant enhancer of PM damage. They validated this using shRNAs, and by rescuing with *HMGCS1*-resistant mutants. Overexpression also showed reduced PM damage after SLO treatment. The authors determined that *HMGCS1* is essential for membrane repair to rescue cell viability following SLO treatment.

To determine if the enzymatic activity of HMGSC1 is essential for membrane repair, Zhang et al. used an enzymatically dead variant to show that it is unable to repair membrane damage following SLO treatment. They also demonstrated that *HMGSC1* depletion significantly decreases cholesterol levels in the cell and cell membrane, mechanistically linking the role of *HMGSC1* in cholesterol synthesis to membrane repair. They orthogonally validated these findings by targeting additional key cholesterol-synthesis genes and providing exogenous, labeled cholesterol molecules to rescue membrane repair. The authors also identify *CHMP4B* localization as a key mediator of *HMGSC1*-dependent membrane repair.

Using several cell lines and *in vivo* models of tumor-immune killing, the authors implicated *HMGCS1* activity in resistance to immune killing via membrane repair. Notably, the authors found that exposure to NK cells drives upregulation of *HMGSC1* in tumor cells. The authors probed the TNF-alpha signaling pathway using small-molecule inhibitors, identifying the transcription factor c-Jun as a key driver of *HMGCS1* expression. They validated this using ChIP and overexpression experiments. Importantly, they showed that c-Jun depletion partially phenocopied *HMGCS1* loss in a CAR-T co-culture assay. Further, they reported that expression of common oncogenic driver mutations known to activate c-Jun increased *HMGCS1* mutation in H1299 cells.

Finally, the authors explored RNA-seq data from the GEO database to examine how *HMGCS1* levels relate to immunotherapy efficacy. They determined that the PD1 non-responders in melanoma have elevated levels of *HMGCS1* RNA. Single-cell data from colorectal cancer (CRC) patients and IF analysis in lung cancer samples showed a similar trend. By stratifying patients into *HMGCS1* high or low groups, they found that high levels of

HMGCS1 are associated with significantly worse patient prognosis. Taken together, the authors argue that *HMGCS1* could be a predictive biomarker of immunotherapy efficacy.

Major points

1. Zhang et al. perform all *in vitro* and *in vivo* studies of *HMCGS1* using A549 and H1299 cells, two models of human lung cancer. However, in Figure 6, they perform analysis of immunotherapy response in other cancer types, including melanoma and colorectal cancer. From these analyses, they suggest that high *HMCGS1* expression may be a more universal immune evasion mechanism. This argument would be substantially strengthened by performing their PMDR assay cells derived from different cancer types, particularly melanoma and CRC. Validation in at least one cell line per tumor type would suffice.
2. In their *in vivo* studies, Zhang et al. demonstrate that knockdown of *HMCGS1* leads to significantly smaller subcutaneous tumors in immunodeficient mice treated by transplantation of NK or CAR-T cells, as well as immunocompetent mice treated with anti-PD-1. While these data are promising, additional pieces of data are requested, as described below.
    a. Despite showing varying volumes at similar timepoints, all *in vivo* experiments appear to end at day 17-19. Survival studies should be conducted to determine whether immunotherapies significantly prolong survival in *HMCGS1* depleted cohorts.
    b. Zhang et al. argue that tumor microenvironment can play a role in elevating *HMCGS1* expression in cancer cells. Orthotopic *in vivo* tumor burden and survival studies in immunocompetent mice with the LLC cancer model with or without immunotherapy are essential in arguing this point.
3. To study the role of the ESCRT complex in promoting membrane repair, the authors focus on the localization of the protein CHMP4B once bound to cholesterol. Through mutagenesis studies, they identify cholesterol recognition motif 2 as a key mediator in binding cholesterol and promoting plasma membrane repair. Analysis of the other core members of the ESCRT complex (e.g. *CHMP2, CHMP3, CHMP6*) for cholesterol binding motifs would further strengthen this point and potentially identify other proteins that could be targeted therapeutically. However, no formal experimental validation of these other possible motifs is requested by this reviewer, but analysis and discussion are recommended.

Minor points

1. Citations for foundational papers identifying the mechanism of perforin pore formation, such as Keefe et al, *Immunity* (2005), should be included.
